# The potential public health impact of Herpes Zoster vaccination in the 65 years of age cohort in Italy

**DOI:** 10.1080/21645515.2019.1657753

**Published:** 2019-09-24

**Authors:** Antonio Volpi, Sara Boccalini, Silvia Dari, Christopher Clarke, Desmond Curran, Idalba Loiacono, Andrea Pitrelli, Anna Puggina, Roberta Tosatto, Desirée Van Oorschot, Elisabetta Franco

**Affiliations:** a Department of Clinical Sciences and Translational Medicine, University of Rome “Tor Vergata”, Rome, Italy; b Department of Health Sciences, University of Florence, Florence, Italy; c NEOS (Neuroscienze, Salute Mentale e Organi di Senso), Sapienza University, Rome, Italy; d Health Economics Zoster, GSK, Wavre, Belgium; e ITA Market Access and Pricing, GSK Italy, Verona, Italy; f Department of Biomedicine and Prevention, University “Tor Vergata”, Rome, Italy

**Keywords:** Herpes zoster, vaccination, public health impact, adjuvanted vaccine, live-attenuated vaccine

## Abstract

Herpes Zoster (HZ) presents a considerable public health burden in Italy among people aged ≥50 years. This study aimed to assess the clinical and economic impact of HZ vaccination in the 65 years of age (YOA) cohort in Italy, by comparing the new Adjuvanted Recombinant Zoster Vaccine (RZV) with the currently available Zoster Vaccine Live (ZVL). A static Markov model was developed to follow all 65 YOA subjects from the year of vaccination over their lifetime by comparing three different HZ vaccination strategies: no vaccination, vaccination with ZVL and vaccination with RZV. In the base-case scenario, three 65 YOA cohorts were assumed to be vaccinated within three years, with a vaccine coverage rate of 20%, 35% and 50% at Year 1, 2 and 3 respectively, as recommended by the National Immunization Plan. The three 65 YOA Italian cohorts accounted altogether for 2,290,340 individuals. Of these, it was assumed that 564,178 subjects could be vaccinated with either RZV or ZVL in three years. The vaccination with RZV could prevent an additional total number of 35,834 HZ and 8,131 postherpetic neuralgia (PHN) cases over ZVL, leading to additional total savings of €12.4 million for the national healthcare and social systems. The introduction of RZV can be expected to have higher impact on the burden of HZ disease in the 65 YOA cohort in Italy. The avoided HZ and PHN cases can lead to an associated reduction in economic burden to the healthcare and social systems.

## Introduction

Herpes Zoster (HZ), commonly known as shingles, is a debilitating disease caused by reactivation of the Varicella Zoster Virus (VZV) that has been dormant in the spinal and cranial sensory ganglia since a primary infection of varicella, that presents itself as chickenpox during childhood.^1,2^ HZ is characterized by a usually painful, unilateral vesicular rash, generally limited to a single dermatome, corresponding to the sensory ganglion from which the latent VZV was reactivated.^3^

In Europe, more than 95% of the adult population show serological signs of a previous VZV infection and are, therefore, at risk of developing HZ.^4^ The main risk factor for reactivation of VZV is represented by the decline in cell-mediated immunity, so that HZ occurs more frequently in older adults and in individuals who are immunocompromised due to underlying disease or immunosuppressive therapy.^5^ In Italy, the overall incidence of HZ has been estimated at 6.42 cases per 1,000 person-years among people aged ≥50 years.^6^

The most common and painful complication of HZ is postherpetic neuralgia (PHN), a chronic neuropathic resilient pain that persists or develops at least 90 days after the acute HZ episode and can continue for months or years.^7^ PHN occurs in 5–30% of patients, but the proportion of patients experiencing it increases with advancing age.^1,2^

HZ and PHN generate a considerable clinical and economic burden in healthcare and socio-economic systems. In Italy, around 157,000 new HZ cases are estimated every year, for total annual costs of €41.2 million (M), of which €28.2M are direct costs and €13.0M are indirect costs.^8,9^

In the US, the vaccination against HZ has become the standard of care for reducing HZ disease burden and complications in older adults,^10^ while the European Center for Disease Prevention and Control (ECDC) reports its recommendation in 6 European countries by 2017.^11^ In Italy, the National Immunization Plan 2017–2019 (NIP) recommends and funds HZ vaccination for all individuals 65 years of age (YOA) and for at-risk subjects (i.e., those affected by diabetes mellitus, cardiovascular diseases, chronic obstructive pulmonary disease, and candidate to immunosuppressive treatment) aged ≥50 YOA.^12^

The Zoster Vaccine Live (ZVL, *Zostavax*, Merck & Co., Inc.), a one-dose vaccine containing the Oka VZV strain, was the first vaccine to be approved for the HZ prevention in adults ≥50 YOA by the Food and Drug Administration (FDA) and the European Medicines Agency (EMA). It was licensed in Italy in 2006.^13,14^

In clinical trials, ZVL has shown an efficacy in preventing HZ and PHN equal to 51.3% and 66.5% respectively in subjects ≥60 YOA,^15^ that decreases with increasing age and wanes over time post vaccination,^16,17^ as also confirmed by the available effectiveness data.^18^ Thus ZVL is suboptimal in preventing HZ in the older age groups who have a higher HZ risk.

Furthermore, as a live-attenuated vaccine, ZVL is contraindicated for use in immunosuppressed or immunodeficient individuals in whom administration of ZVL may result in disseminated disease.^14^

Recently, a new two-dose Adjuvanted Recombinant Zoster Vaccine (RZV, *Shingrix*, GSK) has been developed and approved in Canada, the US and Japan for the prevention of HZ in adults ≥50 YOA, while in Europe and Australia, the license also includes the prevention of PHN.^19–23^

RZV is a non-live vaccine that combines the VZV glycoprotein E (gE) with the AS01_B_, a GSK proprietary Adjuvant System, able to generate VZV-specific, strong, and sustained humoral and cellular immune responses. As a non-live vaccine, there is no contraindication for immunosuppressed patients.^22,24^

In two phase III clinical trials,^24,25^ RZV efficacy against HZ was greater than 90% in all age groups studied with little decline over time up to 4 years. The efficacy of RZV in reducing the PHN and other HZ complications was >90% in all subjects aged ≥50 years.

Based on the available immunogenicity, efficacy, safety and cost-effectiveness data for both ZVL and RZV, in October 2017 the Advisory Committee on Immunization Practices (ACIP) of the Centers for Disease Control and Prevention (CDC, US)^26^ released a preferential recommendation for RZV over ZVL. The ACIP recommended that all the immunocompetent subjects ≥50 YOA should be vaccinated with RZV and the persons who have already received ZVL earlier in life should be revaccinated.^26^ Furthermore, the National Advisory Committee on Immunization (NACI, Canada) has recently released a strong recommendation for using RZV in adults ≥50 YOA for the prevention of HZ without contraindications.^27^

Recent analyses demonstrated the superior public health impact of RZV compared with ZVL in Germany and Japan,^28,29^ as well as the cost-effectiveness of RZV in older adults in USA and Germany.^30,31^

A previous study has shown that introducing a HZ vaccination program for older adults using ZVL would have a beneficial clinical and health economic impact in Italy,^32^ while currently there is no such evidence for RZV in the Italian setting.

The aim of the present study is to evaluate the clinical and related economic impact of implementing an RZV vaccination program in comparison to “no vaccination” and to the current vaccination program with ZVL in the 65 YOA cohort in Italy.

## Materials and methods

### ZOster ecoNomic Analysis model

**Figure 1.**
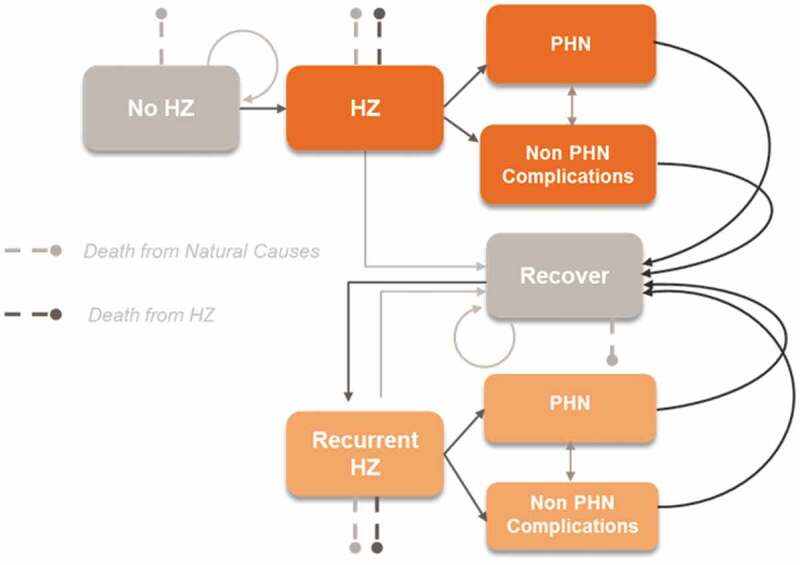
Schematic overview of the ZONA model.^28,29^ HZ: Herpes Zoster; PHN: postherpetic neuralgia. Figure originally published in D. Curran et al. 2017^28^

The ZOster ecoNomic Analysis (ZONA) model was previously developed in MS Excel (Figure 1)^28,29^ and adapted to the Italian healthcare setting to estimate the clinical impact, in terms of HZ and PHN cases, complications and HZ-related deaths avoided, and economic impact, considering both direct and indirect costs avoided, of HZ vaccination in the 65 YOA cohort in Italy. It is a static Markov model that follows the cohort of 65 YOA individuals from the year of vaccination over their lifetime and with annual cycle lengths.

The structure of the model is presented in Figure 1. Health states considered were healthy, HZ, PHN, non PHN complications, and death, as well as recurrent cases. Transition probabilities were derived from Italy-specific literature and are age-specific.

The ZONA model compares three different HZ vaccination strategies: no vaccination (control), vaccination with ZVL, and vaccination with RZV. For each strategy, three cohorts of 65 YOA individuals are assumed to be either vaccinated or not within 3 years with incremental vaccine coverage rates, as recommended by the NIP (i.e., 20% at Year 1, 35% at Year 2, and 50% at Year 3).^12^ The resulting eligible population is presented in Table 1. In the base-case scenario, the compliance to the second dose of RZV was assumed to be 70%. Additionally, for Year 1, two scenario analyses were performed to explore the impact of using alternative RZV second-dose compliance rates, which were assumed to be 50% and 90%, respectively. The same analyses were performed for Years 2 and 3, as presented in the Supplementary Material.

**Table 1.** Three cohorts of Italian subjects 65 YOA eligible to HZ vaccination according to the NIP.^12^

	Cohort 1 – Year 1	Cohort 2 – Year 2	Cohort 3 – Year 3
Population eligible to HZ vaccination			
Individuals 65 YOA^33^	**749,469**	**762,408**	**778,463**
Vaccine coverage rates			
NIP Targets^12^	20%	35%	50%
Population eligible to HZ vaccination based on vaccine coverage rates			
Individuals 65 YOA	149,894	266,483	389,232

YOA: years of age; HZ: Herpes Zoster; NIP: National Immunization Plan.^12^

### Model inputs and assumptions

The model data inputs and assumptions are divided into four sections: demographics, HZ epidemiology, related direct and indirect clinical costs, and vaccine efficacy.

### Demographics

A base-case scenario was assumed for each cohort, as summarized in Table 3. Three cohorts of Italian subjects 65 YOA for the Years 2017 (Year 1), 2018 (Year 2) and 2019 (Year 3) were retrieved from the Italian Institute of Statistics (ISTAT)^33^ counting for approximately 749,000, 762,000 and 778,000 individuals, respectively.

**Table 2.** Base-case epidemiological data inputs.

Variable	Base-case Scenario	Source
**Epidemiological data**		
**HZ Incidence (HZ/1,000 person-years)**		Coretti et al.^8^
65–69 YOA	7.10	
70–79 YOA	8.40	
≥80 YOA	8.40	
**PHN Proportion (%)**		Coretti et al.^8^
65–69 YOA	20.20%	
70–79 YOA	24.50%	
≥80 YOA	24.50%	
**Complications (%)**		Alicino et al.^6^
Ophthalmic HZ	4.00%	
Neurological	0.70%	
Cutaneous	3.70%	
Other	0.00%	

YOA: years of age; HZ: Herpes Zoster; PHN: postherpetic neuralgia.

**Table 3.** Base-case cost data inputs.

Variable	Base-case Scenario	Source
**Costs**		
**Direct costs, €/case**		
HZ	183	Gialloreti et al.*^9^
PHN	616	Gialloreti et al.*^9^
Ophthalmic HZ	197	Ministry of Health^39^
Other	207	Ministry of Health^39^
**Indirect costs, €/case**		Gialloreti et al.*^9^
HZ	612	
PHN	875	

*Cost inflated to 2016 values according to HCHS index; HZ: Herpes Zoster; PHN: postherpetic neuralgia.

### Epidemiological and cost data

Epidemiological data inputs are summarized in Table 2. All-cause mortality rates were derived from the ISTAT database,^34^ while HZ-specific mortality rates were retrieved from the World Health Organization (WHO) database.^35^

The HZ incidence rates and the relative proportion of PHN were extracted from the study of Coretti et al.^8^ In addition to PHN, the analysis included the following complications, derived from Alicino et al.:^6^ ophthalmic HZ (4%), neurological complications (0.7%) and cutaneous complications (0.37%).

Clinical cost data inputs are reported in Table 3. Direct and indirect costs of HZ and PHN were derived from the study of Gialloreti et al.^9^ and were inflated to 2016 prices using the Hospital and Community Health Service (HCHS) index.^36^

### Vaccine efficacy

Vaccine efficacy data points for both RZV and ZVL were previously calculated and validated for a recent similar study performed in Germany by Curran et al.^28^ and were further validated during two advisory boards with Italian experts in epidemiology, health economics and infectious diseases.

As previously described, vaccine efficacy and waning data (i.e. absolute percentage waning over time of efficacy of RVZ in preventing HZ) for both vaccines were derived from the respective randomized controlled trials and are reported in Table S1 (Supplementary Material).

RZV was developed in a two-dose schedule and, from a public health perspective, it is unlikely that second-dose compliance reaches 100%. Consequently, the efficacy of a single dose of RZV was assessed in a post-hoc analysis of the phase III studies by Curran et al.,^28^ as reported in Table S2 (Supplementary Material). Moreover, the RZV single-dose efficacy against HZ was conservatively assumed to wane at 10.9% annually, based on the assumptions made by the ACIP^26^ and as previously published by Le et al.^37^

### Sensitivity analysis

For the Year 1 analysis, a univariate deterministic sensitivity analysis (DSA) was performed with the aim to assess the robustness of the results. The following base-case parameters were varied: HZ incidence rate, PHN proportion, RZV second-dose compliance, vaccine efficacy and waning for both vaccines (ranges are detailed in Table S2 of the Supplementary Material). The corresponding number of HZ cases avoided were recorded and were summarized in a Tornado diagram.

## Results

In the base-case scenario, the three cohorts were assumed to be vaccinated with either RZV or ZVL following the NIP recommendations; the public health impact, in terms HZ and PHN cases, complications and deaths avoided, of the HZ vaccination strategies over lifetime is summarized in Table 4.

**Table 4.** Public health impact of RZV and ZVL in three 65 YOA Italian cohorts under base-case assumptions over a lifetime horizon from the date of vaccination.

	Cohort 1 – Year 1		Cohort 2 – Year 2		Cohort 3 – Year 3		Total Years 1–3
Individuals 65 YOA^33^	749,469		762,408		778,463		2,290,340
Vaccine coverage^12^ and corresponding population^#^	20%	149,894	35%	266,843	50%	389,232	805,968
RZV second-dose compliance and corresponding population^§^	70%	104,926	70%	186,790	70%	272,462	564,178
*Outcomes*	RZV vs ZVL		RZV vs ZVL		RZV vs ZVL		RZV vs ZVL
**Clinical impact of HZ vaccination**							
HZ cases avoided	6,664		11,864		17,306		35,834
PHN cases avoided	1,512		2,692		3,927		8,131
Complications avoided	560		997		1,454		3,011
HZ-related deaths avoided	2		3		5		10
**Economic impact of HZ vaccination (related to clinical savings)**							
Direct costs avoided (€)	1,431,910		2,549,105		3,718,263		7,699,278
Indirect costs avoided (€)	875,431		1,558,453		2,273,245		4,707,129

**Notes**: ^#^receiving ZVL or the first dose of RZV. ^§^receiving the second dose of RZV. YOA: years of age; HZ: Herpes Zoster; PHN: postherpetic neuralgia; RZV: Adjuvanted Recombinant Zoster Vaccine; ZVL: Zoster vaccine Live.

**Figure 2.**
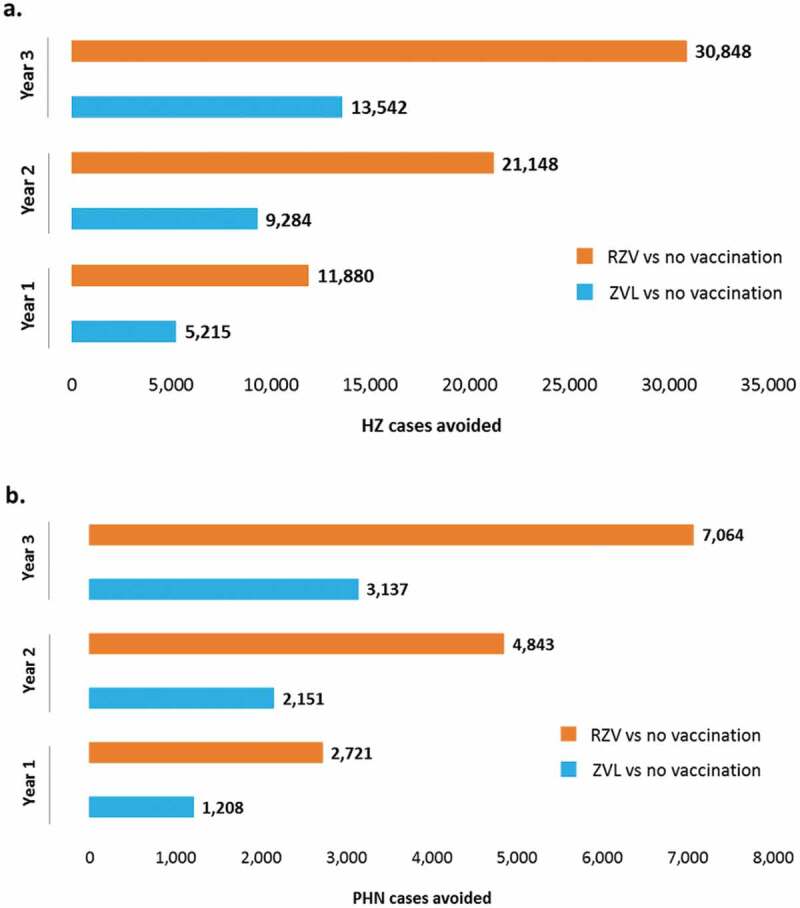
HZ (a) and PHN (b) cases avoided with RZV vs. no vaccination and ZVL vs. no vaccination under base-case assumptions and by year of vaccination. HZ: Herpes Zoster; PHN: postherpetic neuralgia; RZV: Adjuvanted Recombinant Zoster Vaccine; ZVL: Zoster Vaccine Live.

The ZONA model estimated that vaccinating 20% of the first 65 YOA cohort (at Year 1) with RZV would prevent 11,880 HZ cases compared to no vaccination, while 5,215 HZ cases would be avoided if ZVL was used (Figure 2(a)). A two-fold increase in HZ and PHN cases avoidance was demonstrated when vaccinating the further two 65 YOA Italian cohorts at Year 2 and 3 with RZV compared to ZVL. Assuming a coverage rate of 35% at Year 2, it was estimated that RZV would reduce the number of HZ cases by 21,148, compared with 9,284 using ZVL (Supplementary material table S3). At Year 3, vaccinating 50% of the 65 YOA Italian cohort with RZV would avoid 17,306 more HZ cases compared to ZVL.

Similarly, our model predicted that, compared to no vaccination, vaccinating the 65 YOA Italian cohort with RZV would prevent 2,721, 4,843 and 7,064 PHN cases in the three years respectively, compared to 1,208, 2,151 and 3,137 respectively for ZVL (Figure 2(b)).

**Figure 3.**
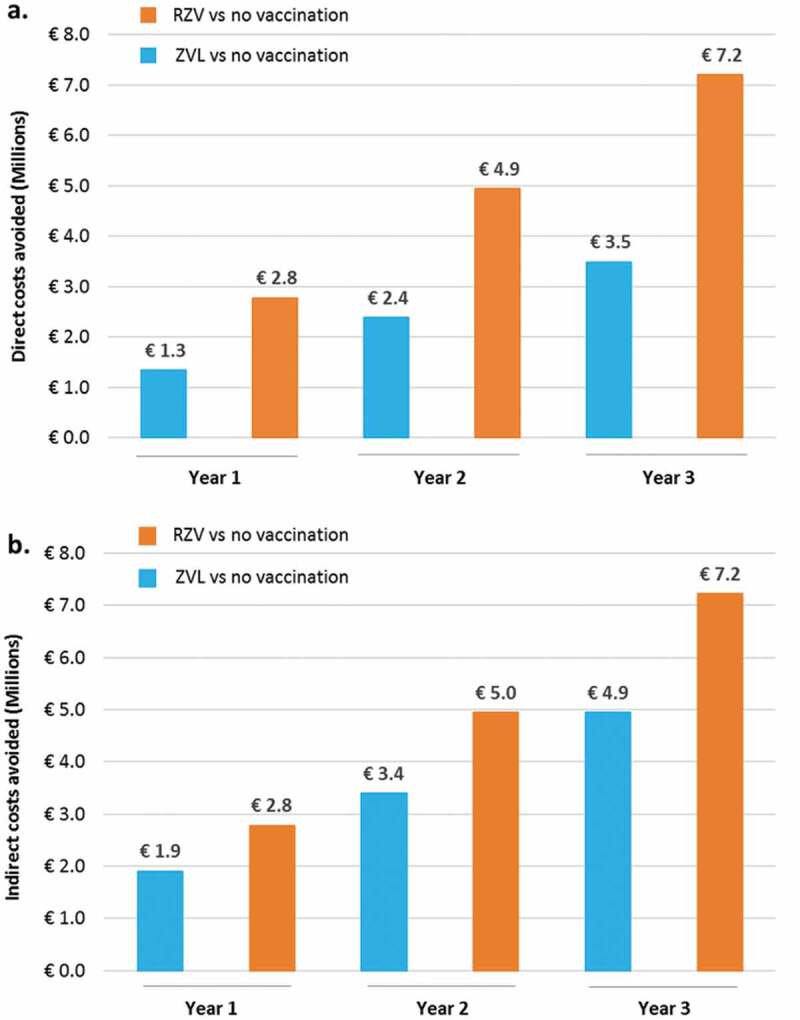
Direct (a) and indirect (b) costs avoided with RZV vs. no vaccination and ZVL vs. no vaccination under base-case assumptions and by year of vaccination. RZV: Adjuvanted Recombinant Zoster Vaccine; ZVL: Zoster Vaccine Live.

Compared to no vaccination, in the base-case scenario, the ZONA model predicted clinical direct costs savings related to HZ, PHN and other complications of €2.8M, €4.9M and €7.2M when vaccinating the 65 YOA Italian cohort with RZV, and of €1.3M, €2.4M and €3.5M using ZVL, at Year 1, 2 and 3, respectively (Figure 3(a)). Indirect costs avoided by preventing HZ, PHN and other complications in the 65 YOA Italian cohort with either RZV or ZVL are also presented in Figure 3(b) and highlight a two-fold additional saving when using RZV compared to ZVL.

**Figure 4.**
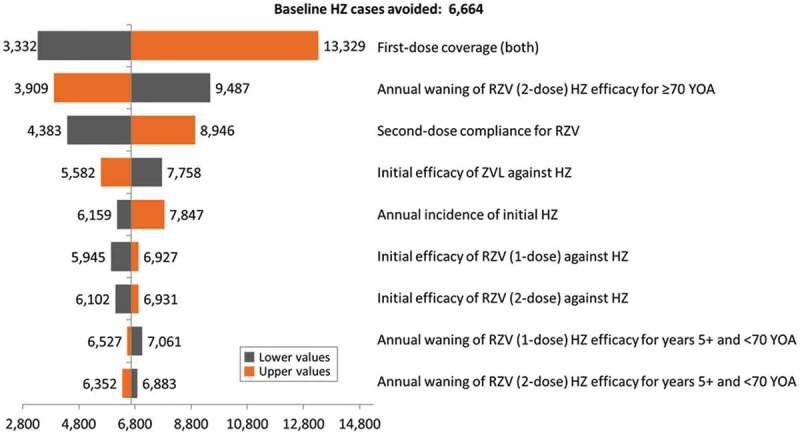
Tornado diagram representing HZ cases avoided with RZV vs. ZVL at year 1 of vaccination. HZ: Herpes Zoster; YOA: years of age; RZV: Adjuvanted Recombinant Zoster Vaccine; ZVL: Zoster Vaccine Live.

Public health impact analysis of RZV and ZVL in three 65 YOA Italian cohorts under base-case assumptions over a lifetime horizon from the date of vaccination revealed that the vaccination with RZV could prevent an additional total number of 35,834 HZ and 8,131 PHN cases over ZVL. This approximately corresponds to a 56% reduction in these outcomes, amounting to a reduction of €12.4M in direct clinical and indirect costs over the three years (Table 4).

Vaccination of the first cohort with RZV (at Year 1, 20% coverage) even considering the worst-case scenario in terms of second-dose compliance (50%), would still prevent 4,383 HZ and 968 PHN cases more than with ZVL, leading to higher direct and indirect costs savings (Table 5). Similar findings were found for Years 2 and 3, as reported in Tables S3 and S4, respectively (Supplementary Material).

**Table 5.** Public health impact of RZV and ZVL in the 65 YOA Italian cohort at Year 1, assuming a coverage rate of 20% over a lifetime horizon from the date of vaccination and different RZV second-dose compliance rates.

	Cohort 1 – Year 1								
Individuals 65 YOA^33^	749,469								
Vaccine coverage^12^ and corresponding population^#^	20%	149,894		20%	149,894		20%	149,894	
RZV second dose compliance and corresponding population^§^	50%	74,947		70%	104,926		90%	134,904	
*Outcomes*	RZV vs no vaccination	ZVL vs no vaccination	RZV vs ZVL	RZV vs no vaccination	ZVL vs no vaccination	RZV vs ZVL	RZV vs no vaccination	ZVL vs no vaccination	RZV vs ZVL
**Clinical impact of HZ vaccination**									
HZ cases avoided	9,598	5,215	4,383	11,880	5,215	6,664	14,161	5,215	8,946
PHN cases avoided	2,176	1,208	968	2,721	1,208	1,512	3,265	1,208	2,057
Complications avoided	806	438	368	998	438	560	1,190	438	751
HZ-related deaths avoided	2	0	2	2	0	2	3	0	3
**Economic impact of HZ vaccination (clinical savings)**									
Direct costs avoided (€)	2,275,227	1,343,742	931,485	2,775,653	1,343,742	1,431,910	3,276,078	1,343,742	1,932,336
Indirect costs avoided (€)	2,573,491	1,905,780	667,712	2,781,211	1,905,780	875,431	2,988,930	1,905,780	1,083,150

**Notes**: ^#^receiving ZVL or the first dose of RZV. ^§^receiving the second dose of RZV. YOA: years of age; HZ: Herpes Zoster; PHN: postherpetic neuralgia; RZV: Adjuvanted Recombinant Zoster Vaccine; ZVL: Zoster Vaccine Live.

The results of the DSA for the 65 YOA Italian cohort at Year 1 are summarized in Figure 4 the Tornado diagram presented in and highlight that in all scenarios, the vaccination with RZV prevents more HZ cases than the vaccination with ZVL. Outcomes of this analysis were most sensitive to the vaccine coverage rates for both vaccines and the RZV second-dose compliance rates.

## Discussion

The aim of this study was to evaluate the potential clinical and economic impact of HZ vaccination (as avoided HZ and PHN cases, complications, HZ-related deaths and related clinical cost savings) in Italian cohort of 65 YOA subjects, as recommended by the NIP, using the new RZV in comparison to no vaccination and to the vaccination with ZVL. The NIP in Italy recommends and funds HZ vaccination for all individuals 65 YOA and for at-risk patients aged ≥50 YOA. This study specifically focuses on the cohort of all individuals 65YOA with the aim to evaluate the potential clinical and economic impact of HZ vaccination, introducing RZV compared to the currently available ZVL.

By using an existing and validated static Markov model that follows all subjects 65 YOA from the year of vaccination over their lifetime and with annual cycle lengths,^28,29^ this public health impact analysis demonstrated the value of both vaccines in reducing the burden of HZ and the related direct clinical and indirect costs for the national healthcare and social systems.

The two main assumptions that led the model to predict superior public health impact in the 65 YOA Italian cohort of RZV compared to ZVL were the higher efficacy and the projections that this efficacy would be sustained over time. Following the NIP recommendations and vaccinating with RZV 20% of the 65 YOA cohort at Year 1, 35% at Year 2, and 50% at Year 3,^12^ a total number of 35,834 HZ and 8,131 PHN cases over ZVL would be prevented. The RZV impact on case avoidance would lead to a substantial favorable economic impact in the management of HZ costs, amounting to an additional reduction of €12.4M in direct clinical and indirect costs over the three years.

A gradual increase of RZV second-dose compliance rates would lead to a higher impact on the number of HZ cases avoided by using RZV rather than ZVL, ranging from approximately 35,834 cases avoided with a 70% compliance rate to 48,102 with a 90% compliance rate, translating in €16.2M additional direct clinical and indirect costs avoided.

The results of this study are consistent with findings previously reported in other countries, demonstrating the higher public health impact of RZV for the prevention of HZ and PHN in older adults compared to no vaccination or vaccination with ZVL.^28,29^ Moreover, the ZONA model provided public health impact results that are in line with those obtained in cost-effectiveness analyses of HZ vaccination in older adults performed in the US and Germany.^30,31^ The latter is in turn consistent with studies that used other independent models.^26,37^ The results of these studies proved the higher public health impact of RZV compared to ZVL, which has been further recognized and transformed into a recommendation for the use of RZV in the US,^26^ Canada^27^ and Germany.^38^

The robustness of the ZONA model was supported by several other publications,^28–31^ and data input and assumptions were confirmed by Italian experts in epidemiology, health economics and infectious diseases in 2 Advisory Boards. The major strength of the present study is that its scenarios were developed based on the current NIP recommendations by the Italian Ministry of Health, thus allowing a concrete contextualization of the results obtained. Moreover, this study included numerous local data sources that ensured an accurate estimation of the potential public health impact of HZ vaccination in the Italian setting. A full health economic analysis is planned, which includes the cost-effectiveness and a complete sensitivity analysis of introducing RZV in the Italian setting.

However, this study has some limitations. First, as long-term RZV waning rates are not yet available, we have used the same assumptions as in the impact analysis performed for Germany,^28^ in which both the vaccine efficacy waning rates and upper and lower bounds were validated with a group of international experts. Furthermore, the RZV single-dose efficacy against HZ was conservatively assumed to wane at 10.9% annually, based on the assumptions made by the ACIP^26^ and as previously published by Le et al.^37^

A second limitation of this analysis is the current lack of data on RZV effectiveness in the real-world setting. Third, the RZV second-dose compliance had to be assumed as data recorded in both the ZOE-50 and ZOE-70 studies (~95%) were not applicable in the real-world setting; therefore, scenario analyses were performed to assess the impact of its variation from 50% to 90%. Nevertheless, even in the worst-case scenario we considered in this analysis (50%), vaccinating with RZV would still prevent more HZ and PHN cases than ZVL, leading to higher direct clinical and indirect costs savings.

Currently, there is no price available for RZV in Italy. Therefore, this study focused on the potential clinical benefits and associated cost avoidance due to prevention by vaccination and does not take into account investment costs. A full assessment of the cost effectiveness of HZ vaccination will not be possible until RZV price becomes available.

Thirdly, the NIP recommends vaccinating both the 65YOA cohort and ≥50 YOA with an at-risk condition. Since there is no robust data available for these at-risk conditions we were unable to do a full analysis to demonstrate the overall impact of HZ vaccination within the NIP setting. For future research it would be an added benefit to have such data available. A full health economic analysis is planned, which includes the cost-effectiveness and a complete sensitivity analysis of introducing RZV in the Italian setting.

Based on this analysis, it can be expected that RZV would have a higher clinical impact compared to ZVL on the burden of HZ disease in Italy when introduced into the NIP. The avoided HZ and PHN cases would be associated with a reduction in healthcare resource utilization and associated costs. This evidence may help policy makers and clinicians to make a more informed decision about HZ vaccination.

## Highlights/Focus on patient section

***What is the context?***

Herpes Zoster (HZ), also known as shingles, mostly occurs in adults aged ≥50 years and with a weakened immune system. HZ is characterized by a painful rash that can be followed by a long-lasting pain, a complication defined as postherpetic neuralgia (PHN). In Italy, this disease affects about 6/1,000 person-years among adults aged ≥50 years, translating in around 157,000 new HZ cases per year. Due to the high number of cases and treatment associated, HZ has a high impact on the Italian healthcare and socio-economic systems.

HZ can be prevented through vaccination, that is now recommended by the 2017–2019 Italian National Immunization Plan for everyone aged 65 years and for subjects aged ≥50 years at risk for presence of chronic obstructive pulmonary disease, diabetes, cardiovascular disease or because candidate to immunosuppressive therapy. A one-dose Zoster Vaccine Live (ZVL) is already available in Italy but recently a new two-dose Adjuvanted Recombinant Zoster Vaccine (RZV) has been approved in Europe.

***What is new?***

While the impact of the ZVL was already assessed in Italy, this is the first evaluation of the potential public health impact (in terms of avoided HZ and PHN cases and clinical costs) of the new RZV in Italy using a well-validated mathematical model that compared three different strategies:

- no vaccination
- vaccination with ZVL
- vaccination with RZV.

In our projections, vaccinating 3 cohorts of individuals aged 65 years with RZV in Italy would have a greater impact on reducing the burden of HZ, with 35,834 HZ and 8,131 PHN additional cases avoided, compared with the vaccination with ZVL.

***What is the impact?***

HZ vaccination offers the potential to substantially reduce the HZ burden in Italy. Our model predicts that RZV prevents more HZ and PHN cases than ZVL, with also a major reduction of disease-related costs thus translating into a higher positive impact for the Italian healthcare system.

## Trademark

Shingrix is a trademark owned by or licensed to the GSK group of companies.

Zostavax is a trademark from Merck Sharp & Dohme Corp.
