# Supplementary material for: The potential public health impact of Herpes Zoster vaccination in the 65 years of age cohort in Italy
Source: Hum Vaccin Immunother. 2019 Sep 24;16(2):327–34. doi: 10.1080/21645515.2019.1657753 (PMC7062451; doi:10.1080/21645515.2019.1657753)
Supplement: Supplemental Material [file khvi-16-02-1657753-s001.zip › Slides - HE_PHI Ms_Italy.pptx]

## Slide 1
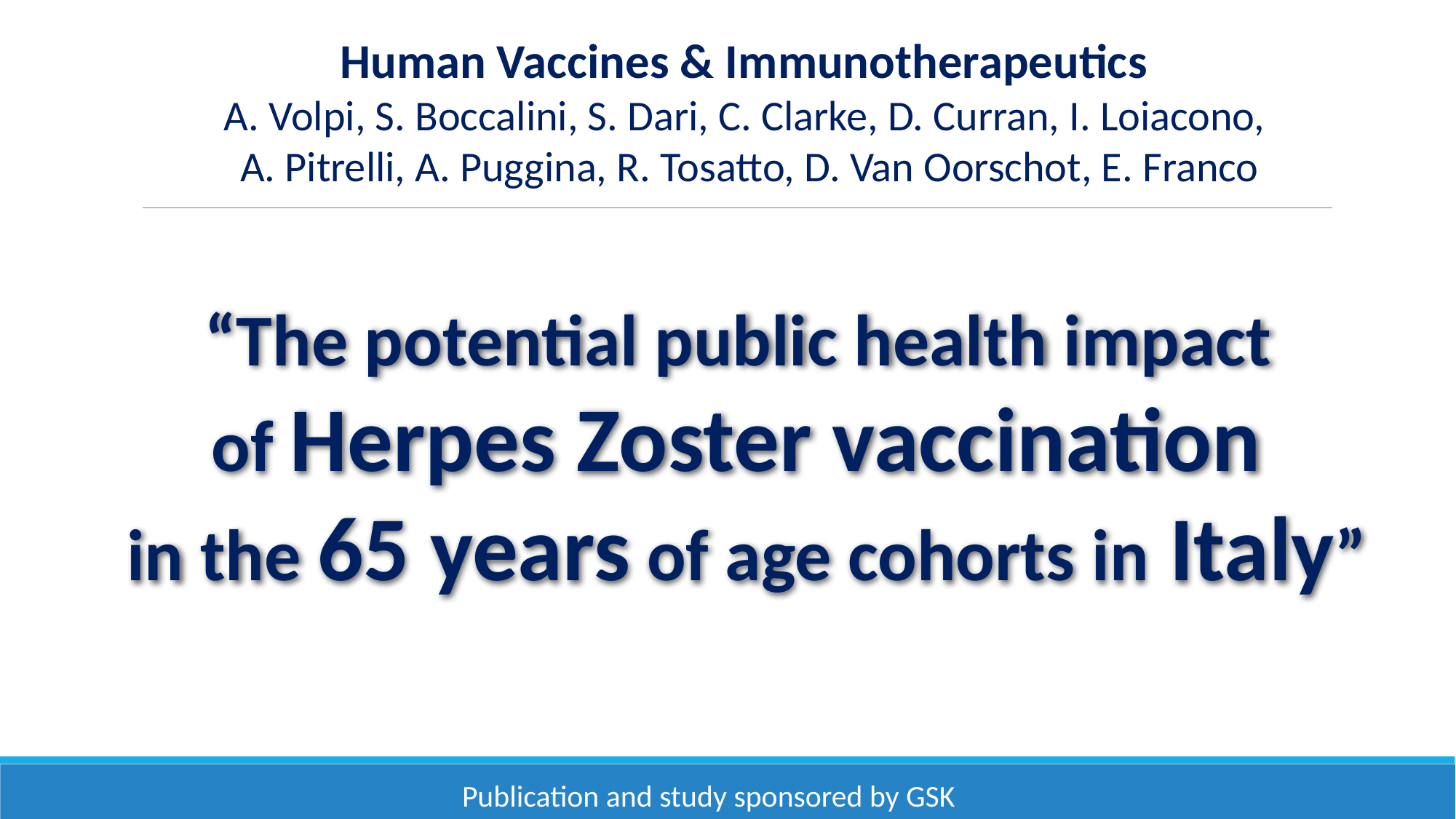

Human Vaccines & Immunotherapeutics
A. Volpi, S. Boccalini, S. Dari, C. Clarke, D. Curran, I. Loiacono,
A. Pitrelli, A. Puggina, R. Tosatto, D. Van Oorschot, E. Franco
“The potential public health impact
of Herpes Zoster vaccination
in the 65 years of age cohorts in Italy”
Publication and study sponsored by GSK

## Slide 2
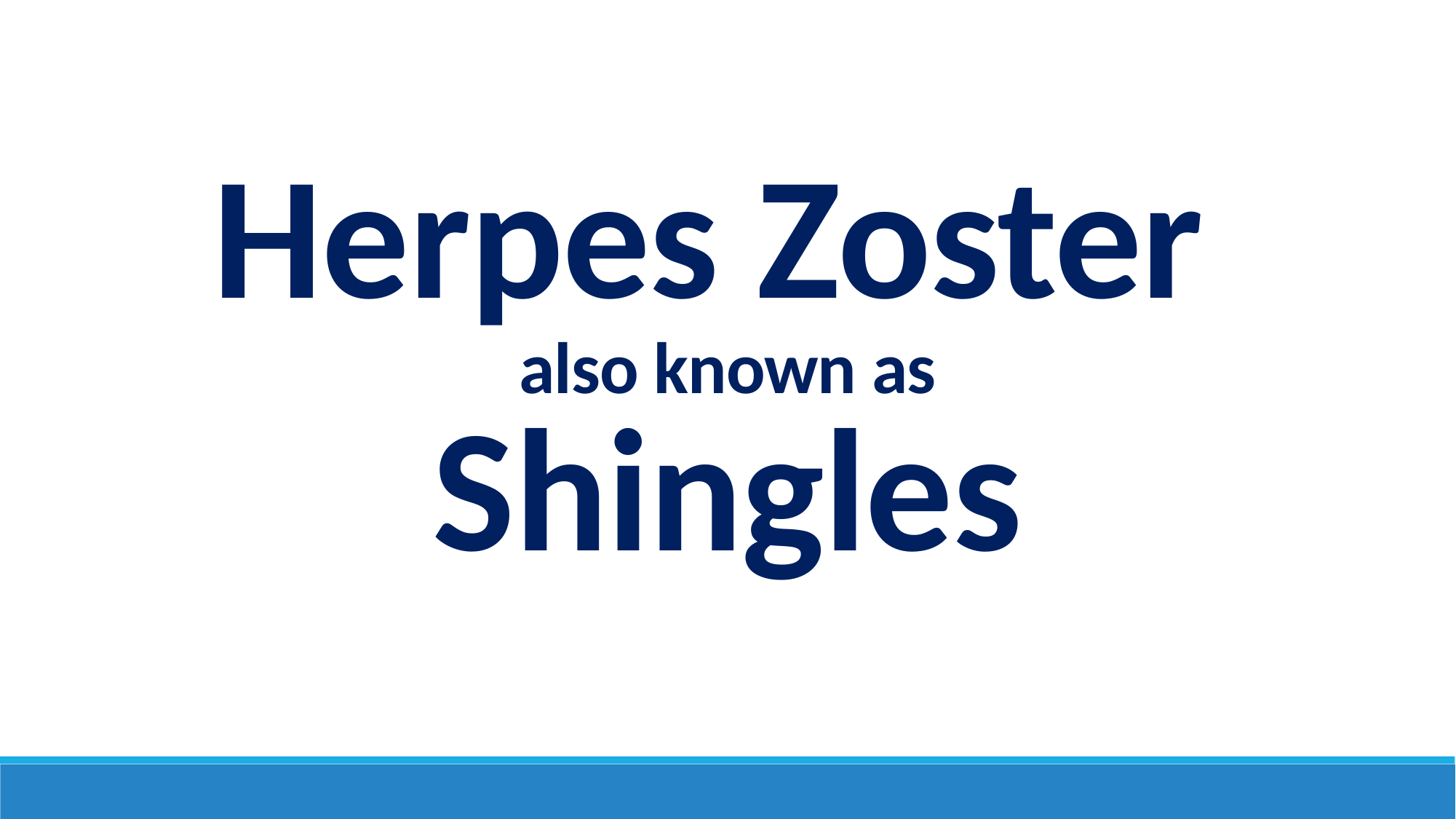

Herpes Zoster
also known as
Shingles

## Slide 3
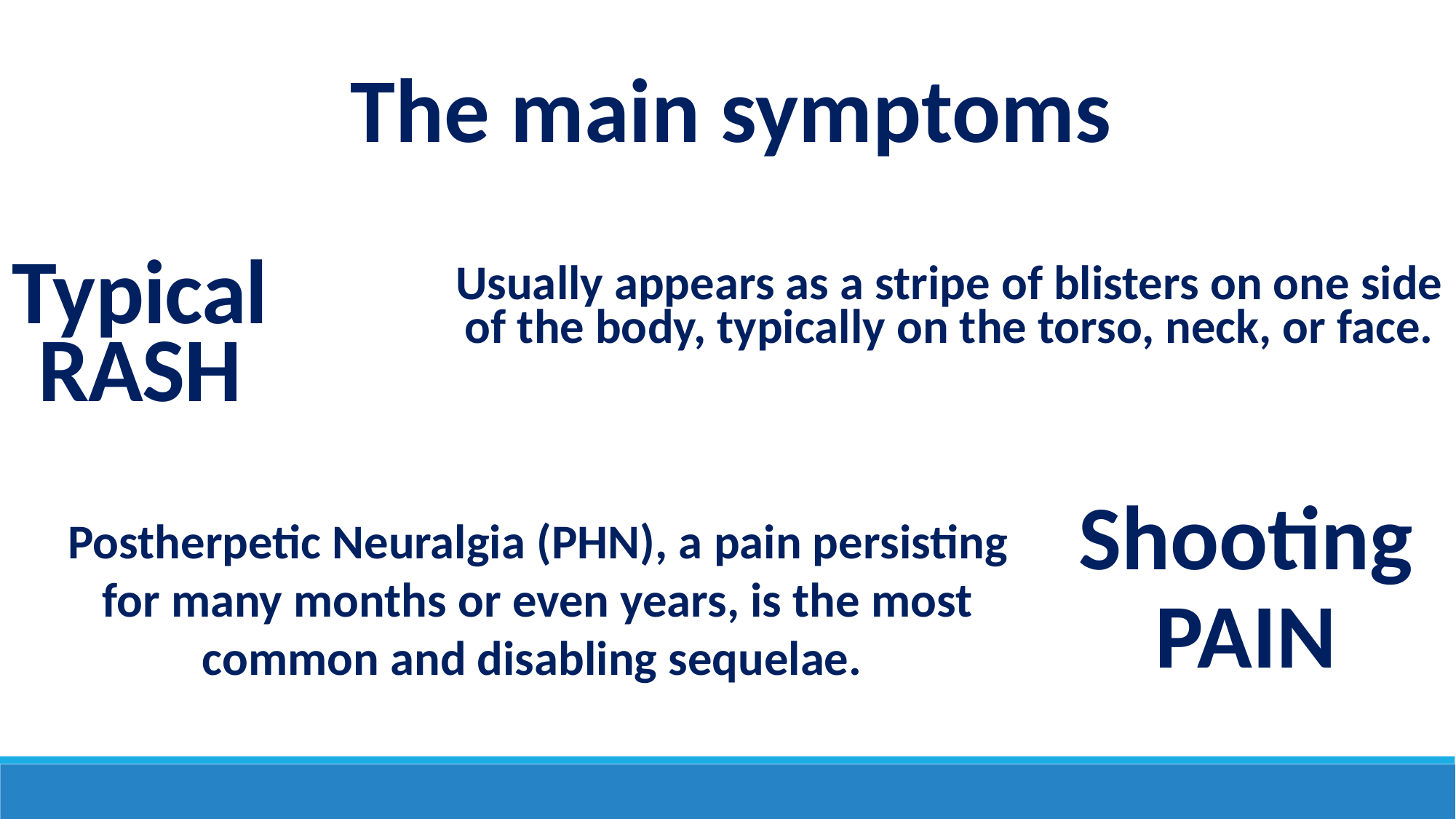

The main symptoms
Typical
RASH
Usually appears as a stripe of blisters on one side of the body, typically on the torso, neck, or face.
Shooting PAIN
Postherpetic Neuralgia (PHN), a pain persisting for many months or even years, is the most common and disabling sequelae.

## Slide 4
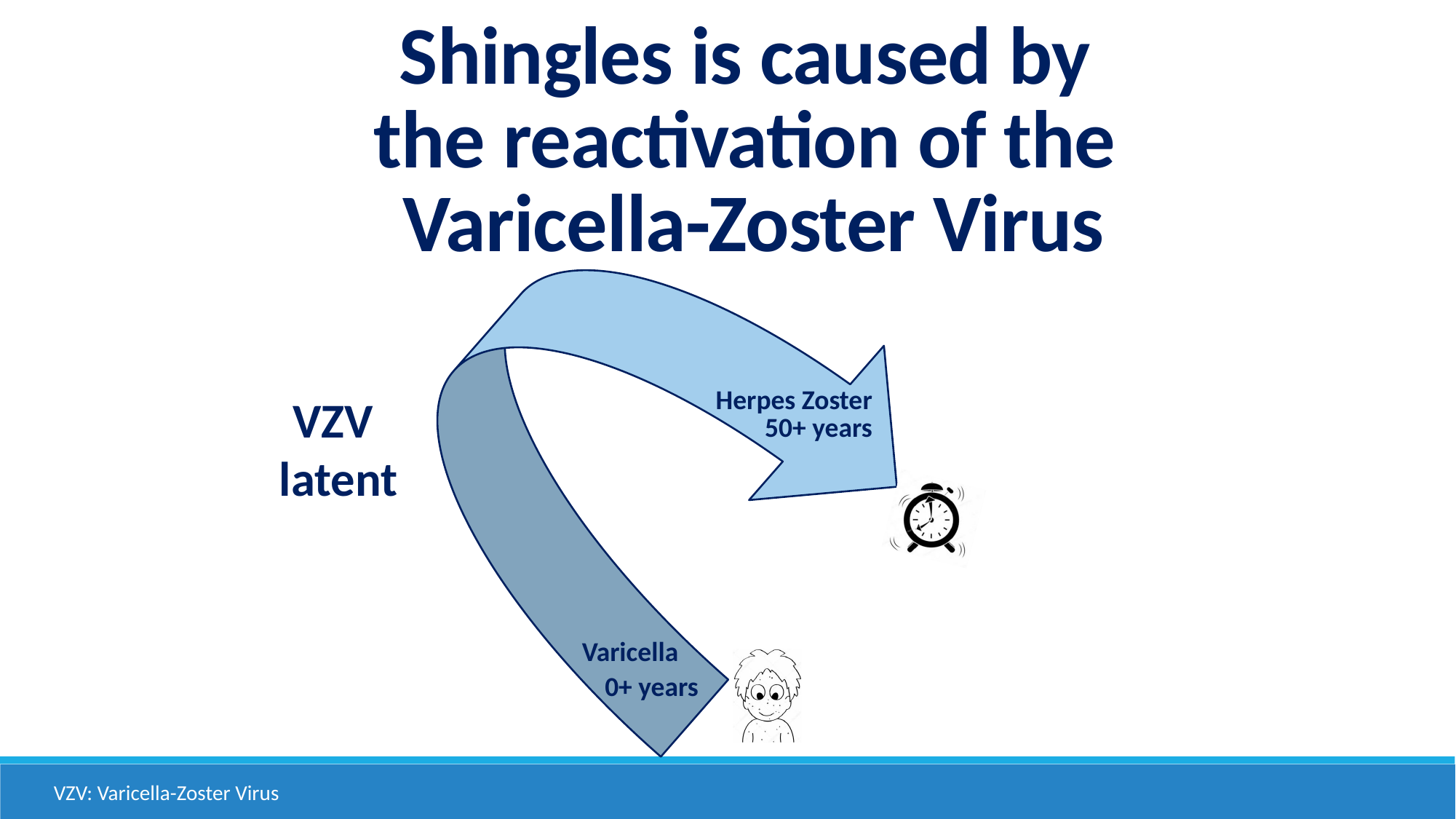

Shingles is caused by
the reactivation of the
Varicella-Zoster Virus
Herpes Zoster
VZV latent
50+ years
Varicella
0+ years
VZV: Varicella-Zoster Virus

## Slide 5
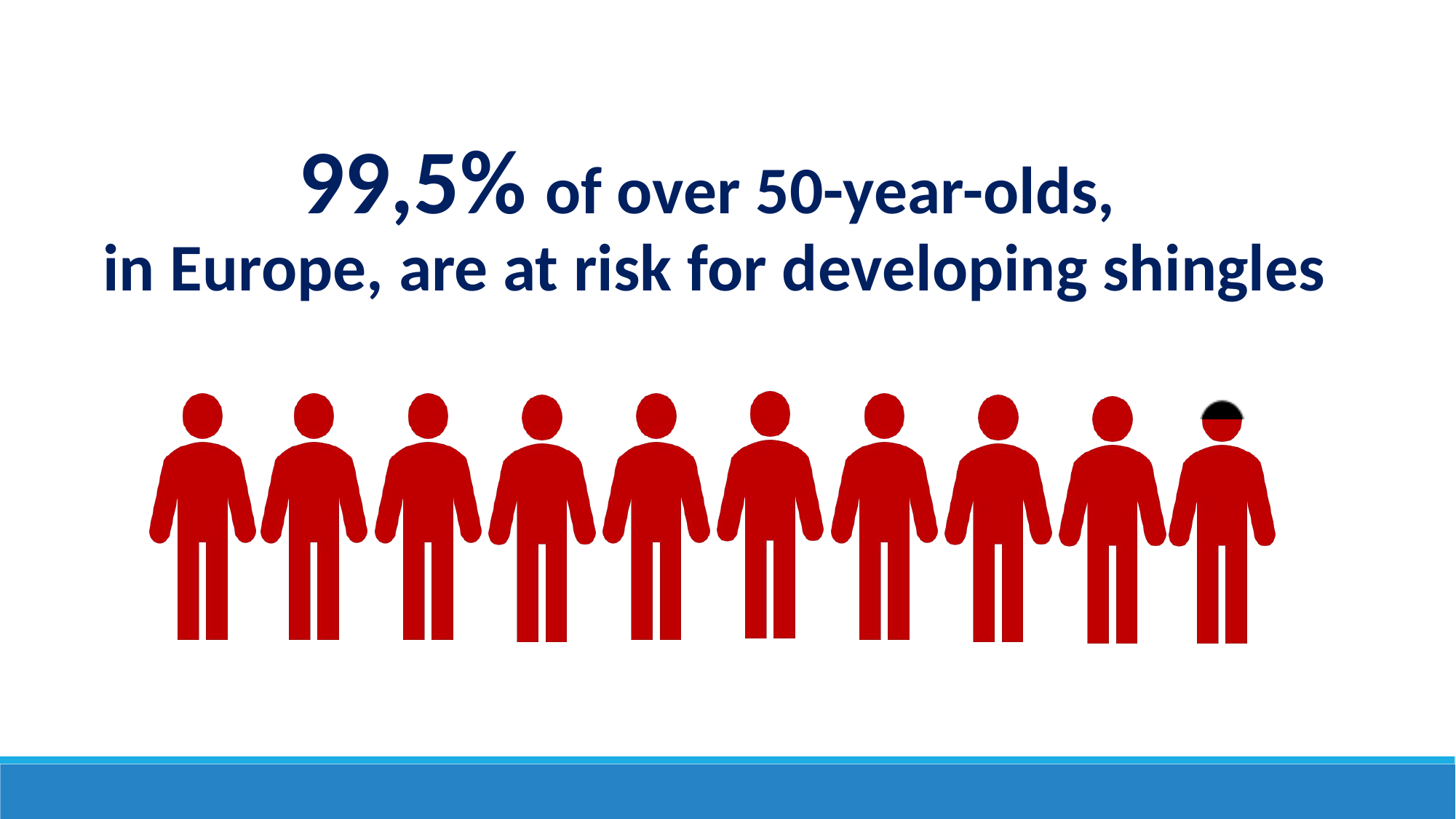

99,5% of over 50-year-olds,
in Europe, are at risk for developing shingles

## Slide 6
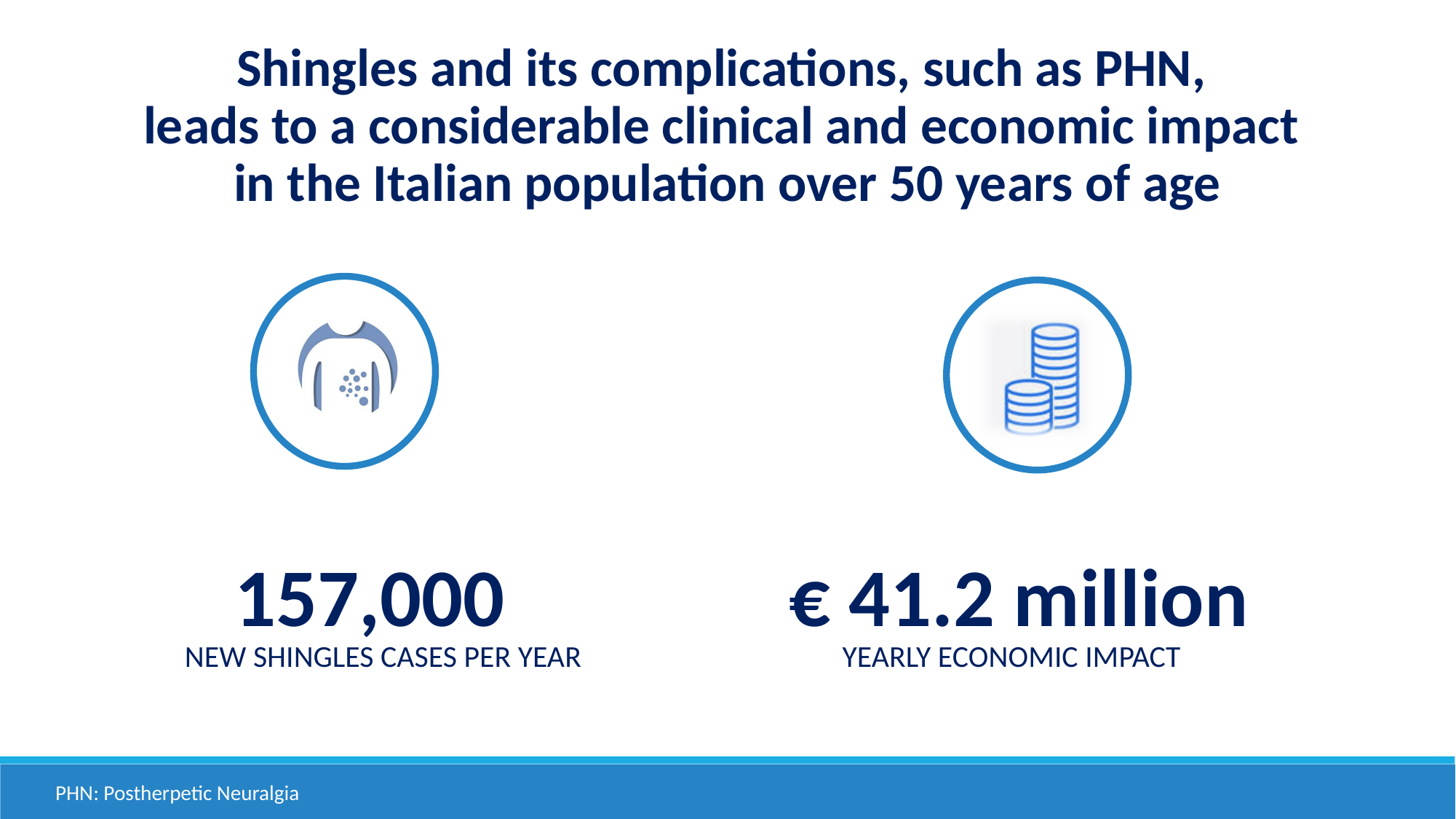

Shingles and its complications, such as PHN,
leads to a considerable clinical and economic impact
in the Italian population over 50 years of age
157,000
€ 41.2 million
NEW SHINGLES CASES PER YEAR
YEARLY ECONOMIC IMPACT
PHN: Postherpetic Neuralgia

## Slide 7
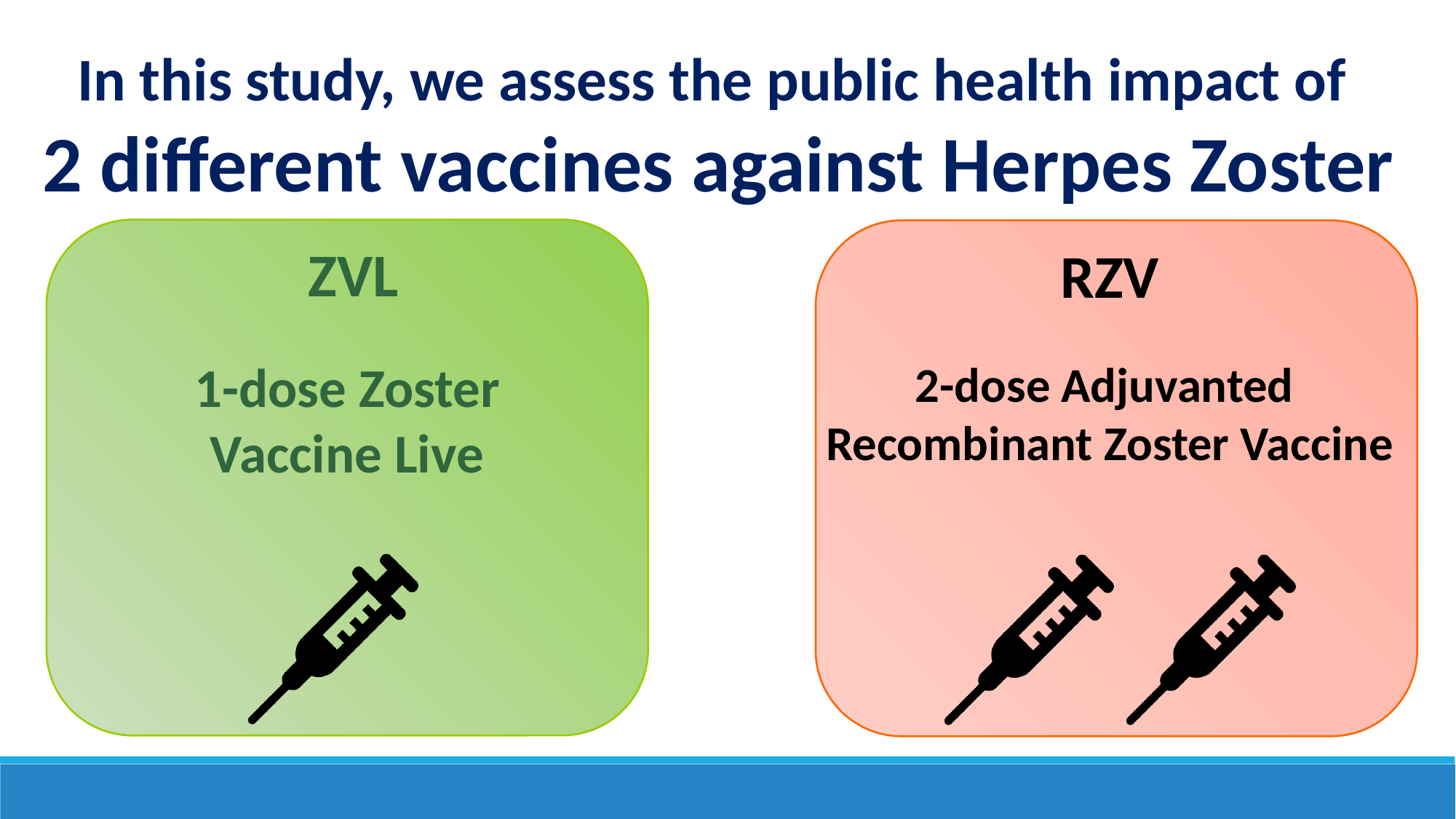

In this study, we assess the public health impact of
2 different vaccines against Herpes Zoster
ZVL
1-dose Zoster
Vaccine Live
RZV
2-dose Adjuvanted
Recombinant Zoster Vaccine

## Slide 8
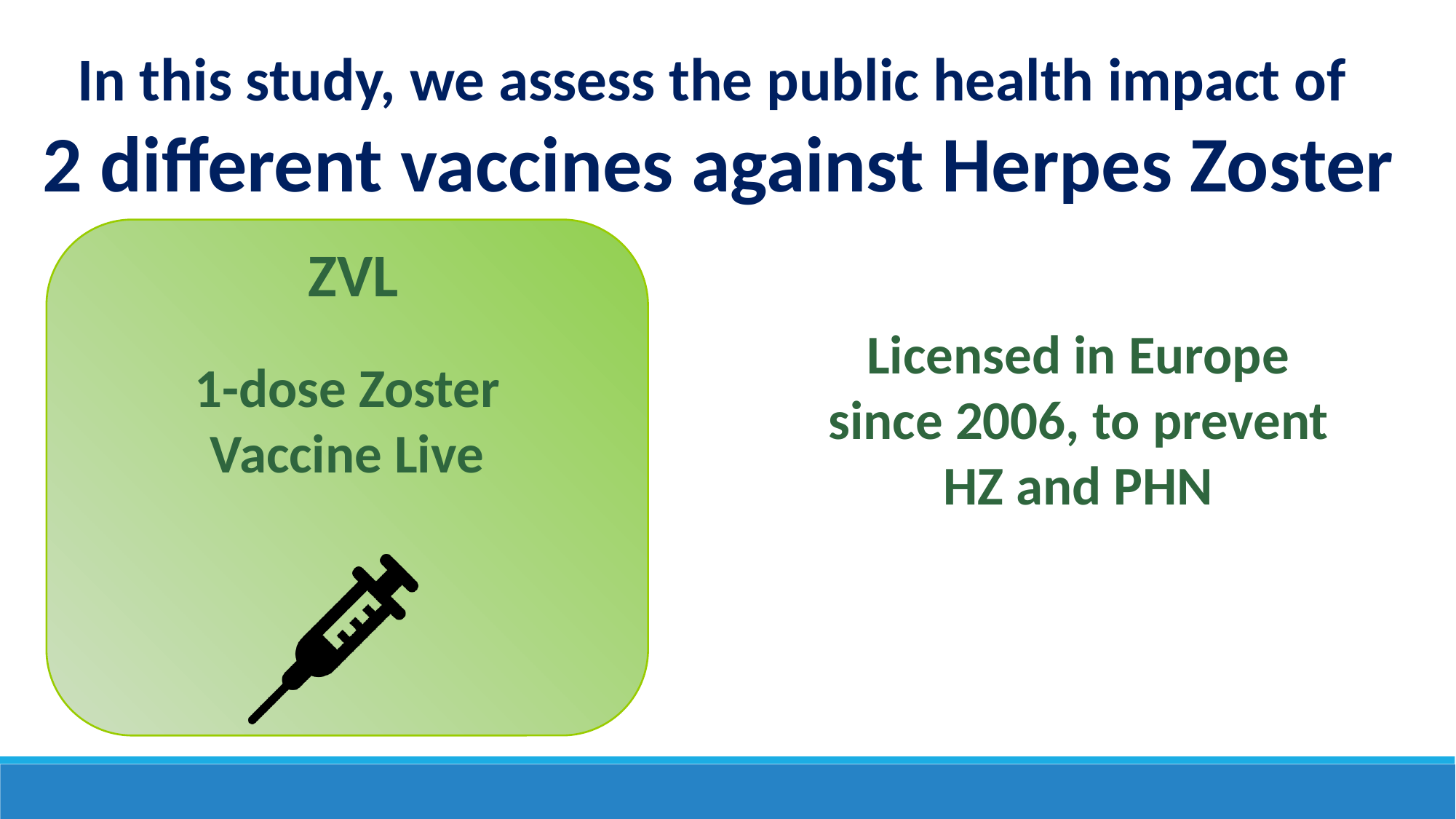

In this study, we assess the public health impact of
2 different vaccines against Herpes Zoster
ZVL
1-dose Zoster
Vaccine Live
Licensed in Europe since 2006, to prevent HZ and PHN

## Slide 9
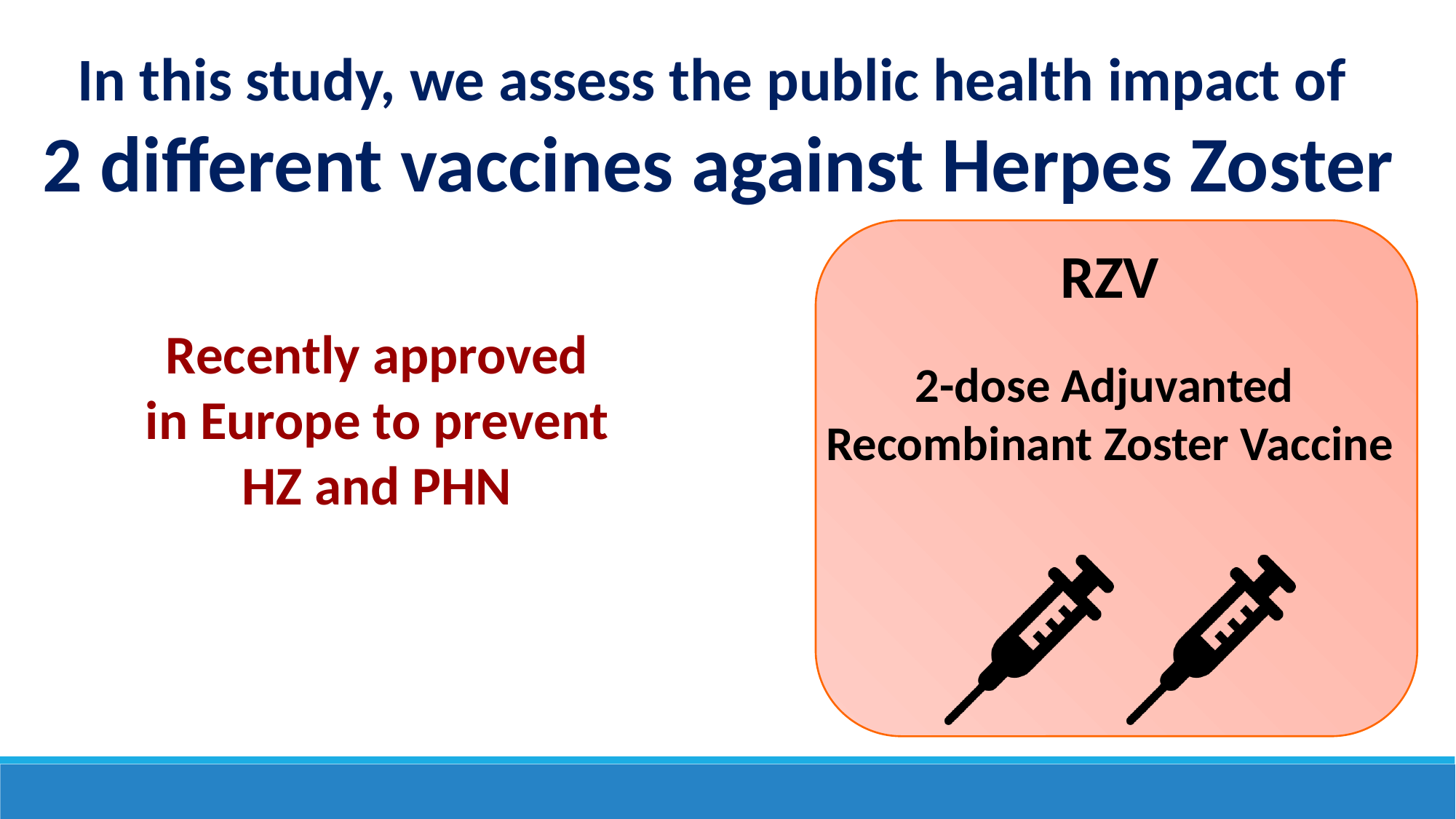

In this study, we assess the public health impact of
2 different vaccines against Herpes Zoster
RZV
2-dose Adjuvanted
Recombinant Zoster Vaccine
Recently approved in Europe to prevent HZ and PHN

## Slide 10
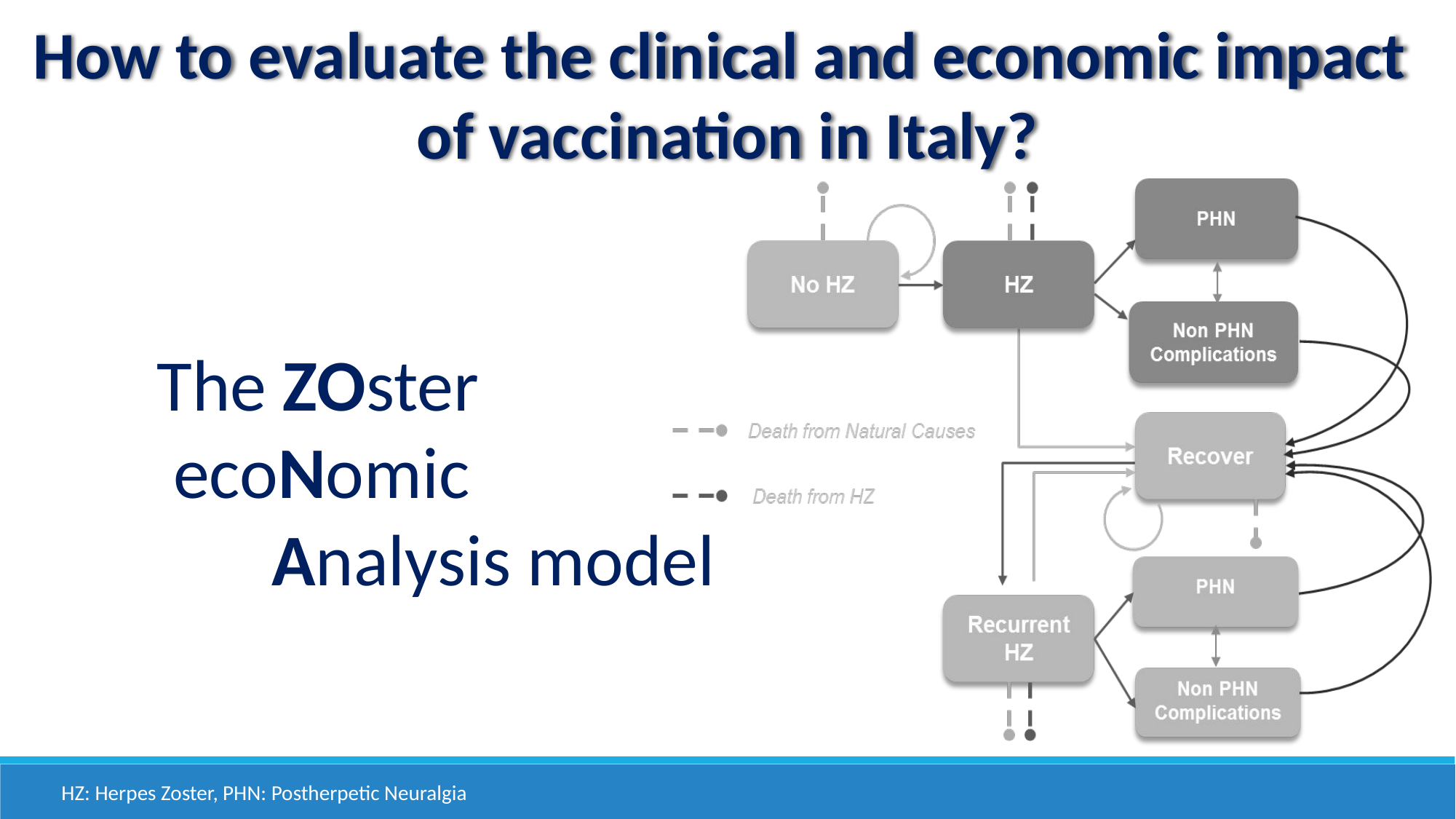

How to evaluate the clinical and economic impact
of vaccination in Italy?
The ZOster
 ecoNomic
 Analysis model
HZ: Herpes Zoster, PHN: Postherpetic Neuralgia

## Slide 11
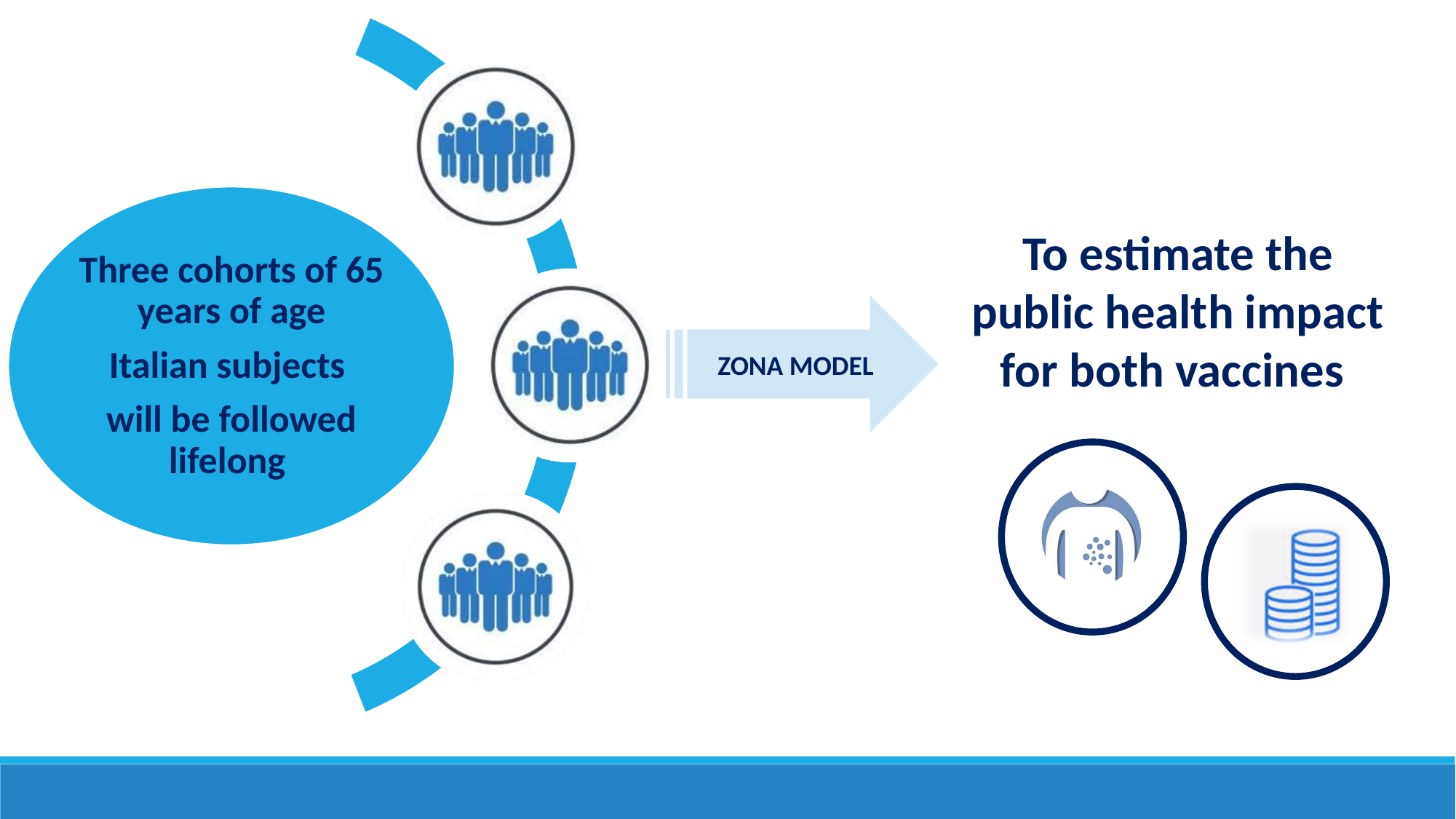

To estimate the
public health impact
for both vaccines
ZONA MODEL

## Slide 12
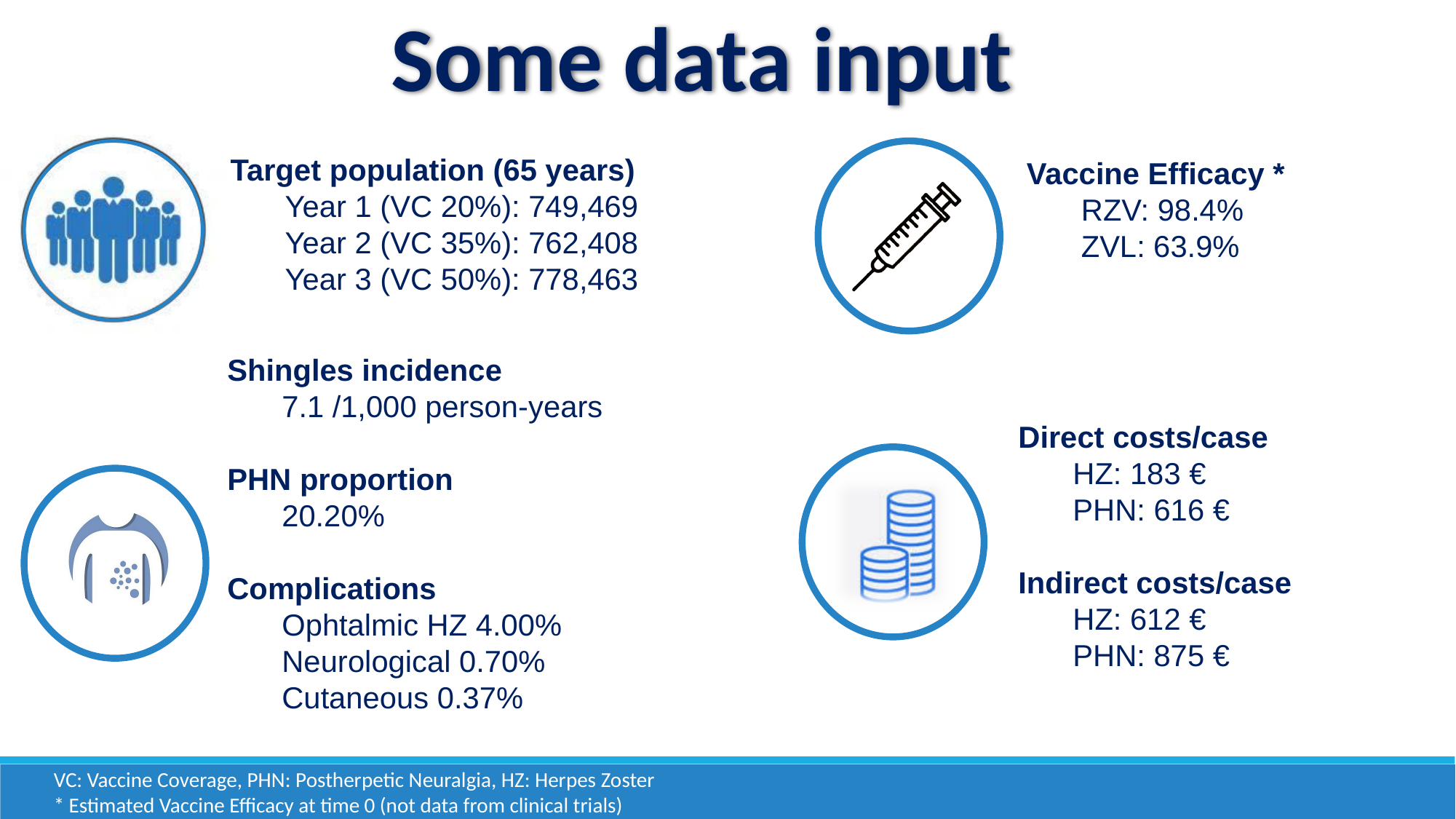

Some data input
Target population (65 years)
Year 1 (VC 20%): 749,469
Year 2 (VC 35%): 762,408
Year 3 (VC 50%): 778,463
Vaccine Efficacy *
RZV: 98.4%
ZVL: 63.9%
Shingles incidence
7.1 /1,000 person-years
PHN proportion
20.20%
Complications
Ophtalmic HZ 4.00%
Neurological 0.70%
Cutaneous 0.37%
Direct costs/case
HZ: 183 €
PHN: 616 €
Indirect costs/case
HZ: 612 €
PHN: 875 €
VC: Vaccine Coverage, PHN: Postherpetic Neuralgia, HZ: Herpes Zoster
* Estimated Vaccine Efficacy at time 0 (not data from clinical trials)

## Slide 13
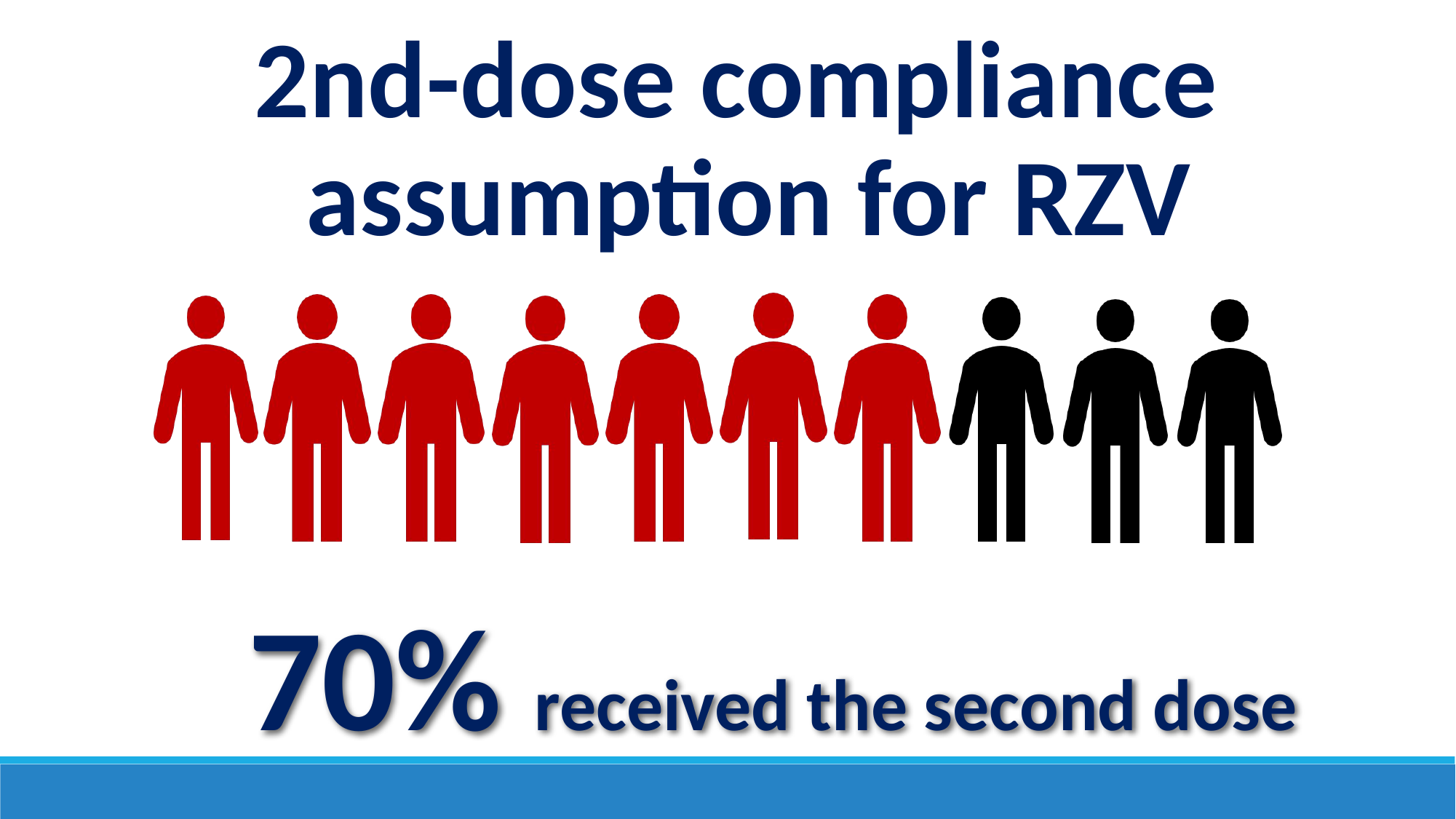

2nd-dose compliance
 assumption for RZV
70% received the second dose

## Slide 14
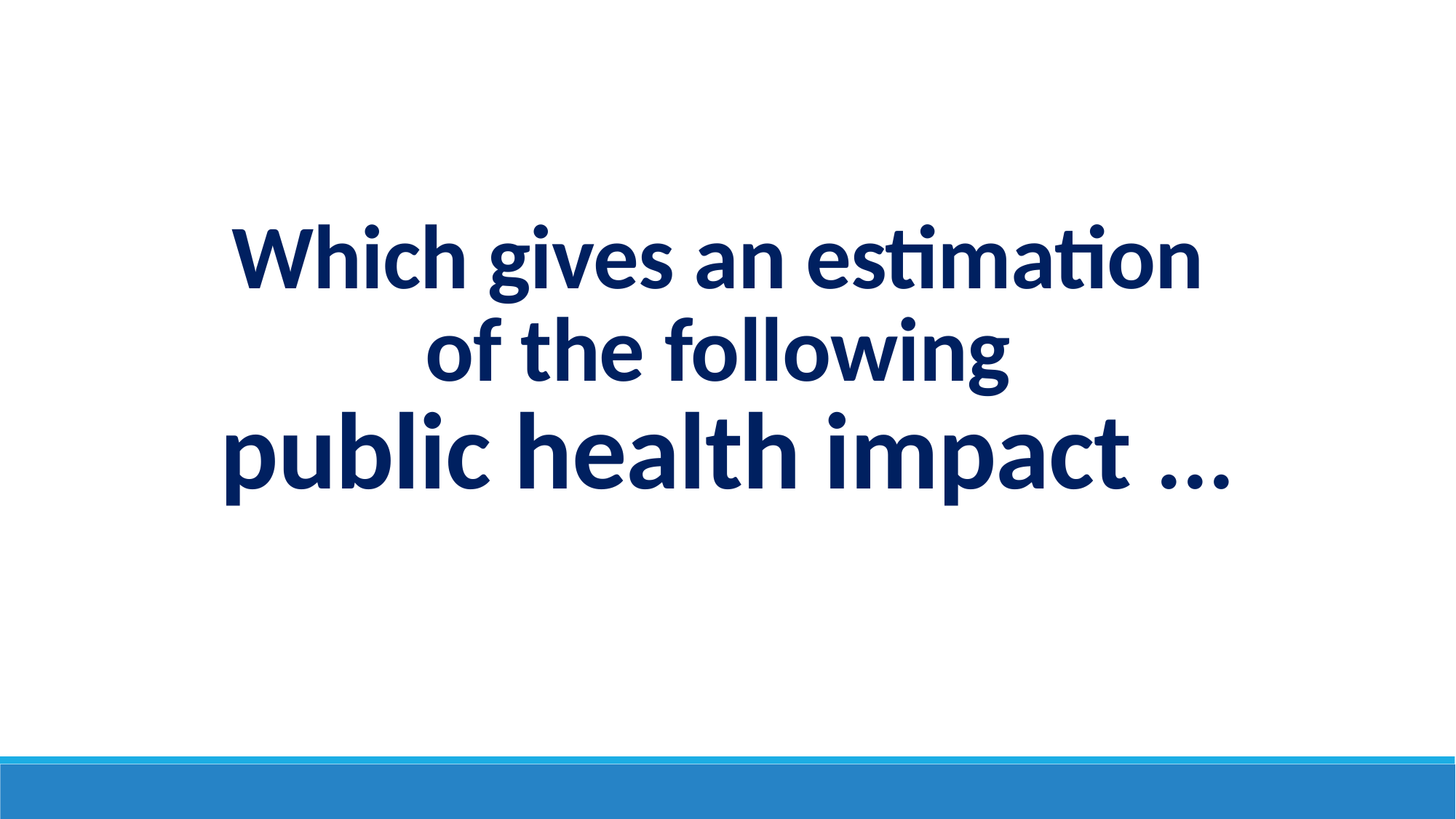

Which gives an estimation
of the following
public health impact ...

## Slide 15
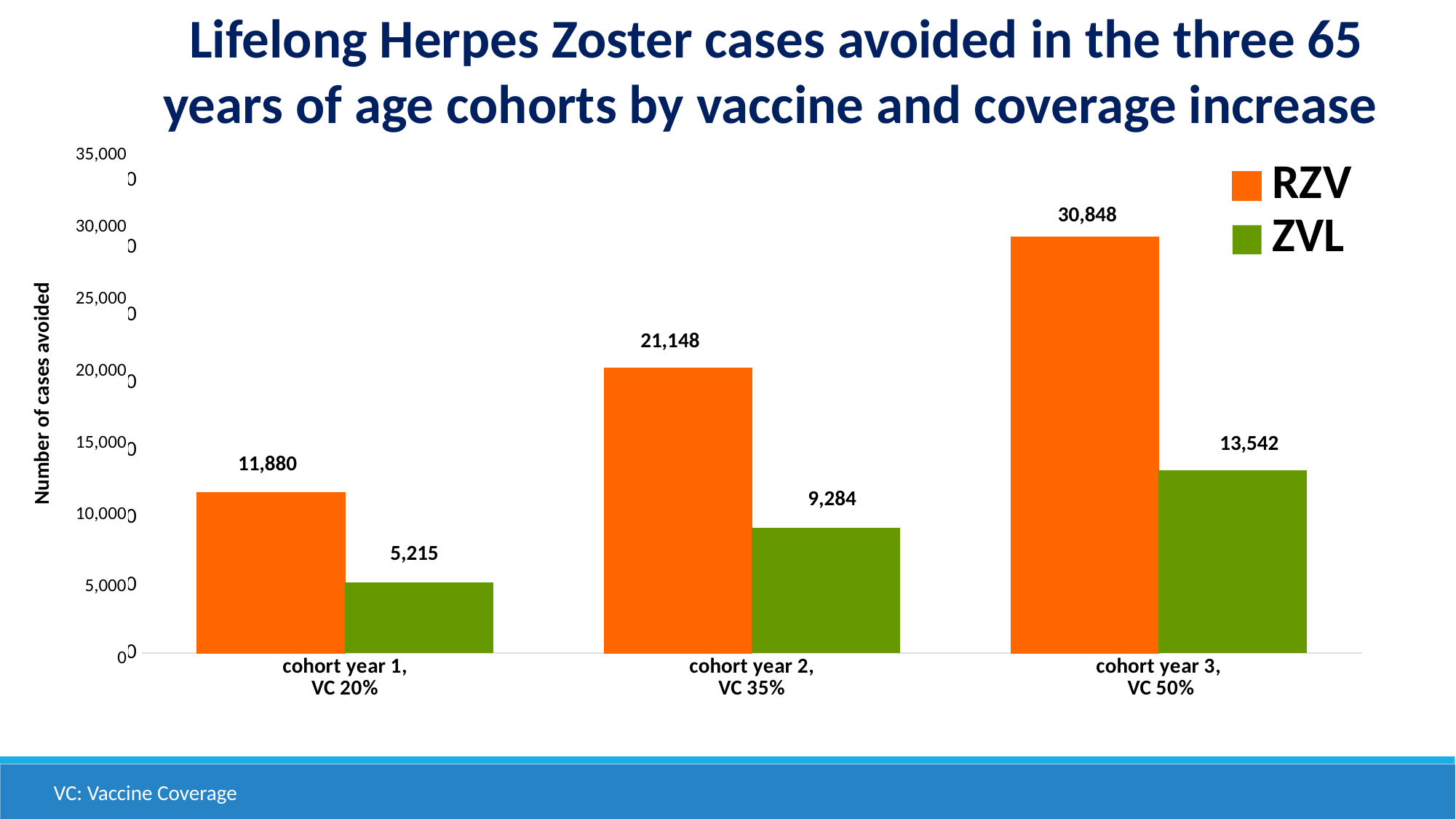

Lifelong Herpes Zoster cases avoided in the three 65 years of age cohorts by vaccine and coverage increase
| 35,000 |
| --- |
| |
| |
| 30,000 |
| |
| |
| 25,000 |
| |
| |
| 20,000 |
| |
| |
| 15,000 |
| |
| |
| 10,000 |
| |
| |
| 5,000 |
| |
| |
| 0 |
### Chart
| Category | RZV | ZVL |
|---|---|---|
| cohort year 1,
VC 20% | 11880.0 | 5215.0 |
| cohort year 2,
VC 35% | 21148.0 | 9284.0 |
| cohort year 3,
 VC 50% | 30848.0 | 13542.0 |30,848
21,148
Number of cases avoided
13,542
11,880
9,284
5,215
VC: Vaccine Coverage
15

## Slide 16
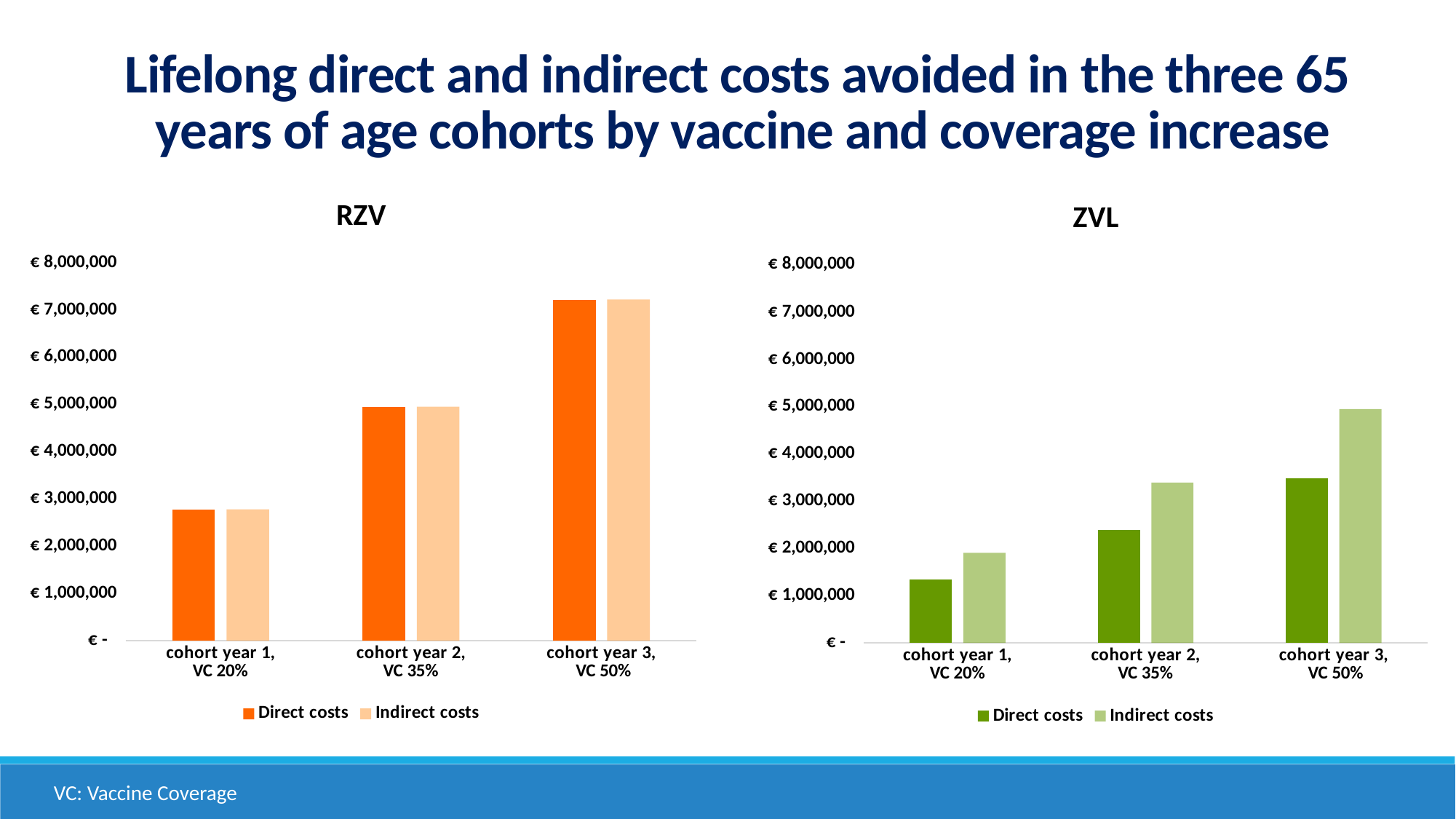

Lifelong direct and indirect costs avoided in the three 65
years of age cohorts by vaccine and coverage increase
### Chart: RZV
| Category | Direct costs | Indirect costs |
|---|---|---|
| cohort year 1,
VC 20% | 2775653.0 | 2781211.0 |
| cohort year 2,
VC 35% | 4941251.0 | 4951145.0 |
| cohort year 3,
 VC 50% | 7207579.0 | 7222012.0 |
### Chart: ZVL
| Category | Direct costs | Indirect costs |
|---|---|---|
| cohort year 1,
VC 20% | 1343742.0 | 1905780.0 |
| cohort year 2,
VC 35% | 2392147.0 | 3392693.0 |
| cohort year 3,
 VC 50% | 3489316.0 | 4948767.0 |VC: Vaccine Coverage

## Slide 17
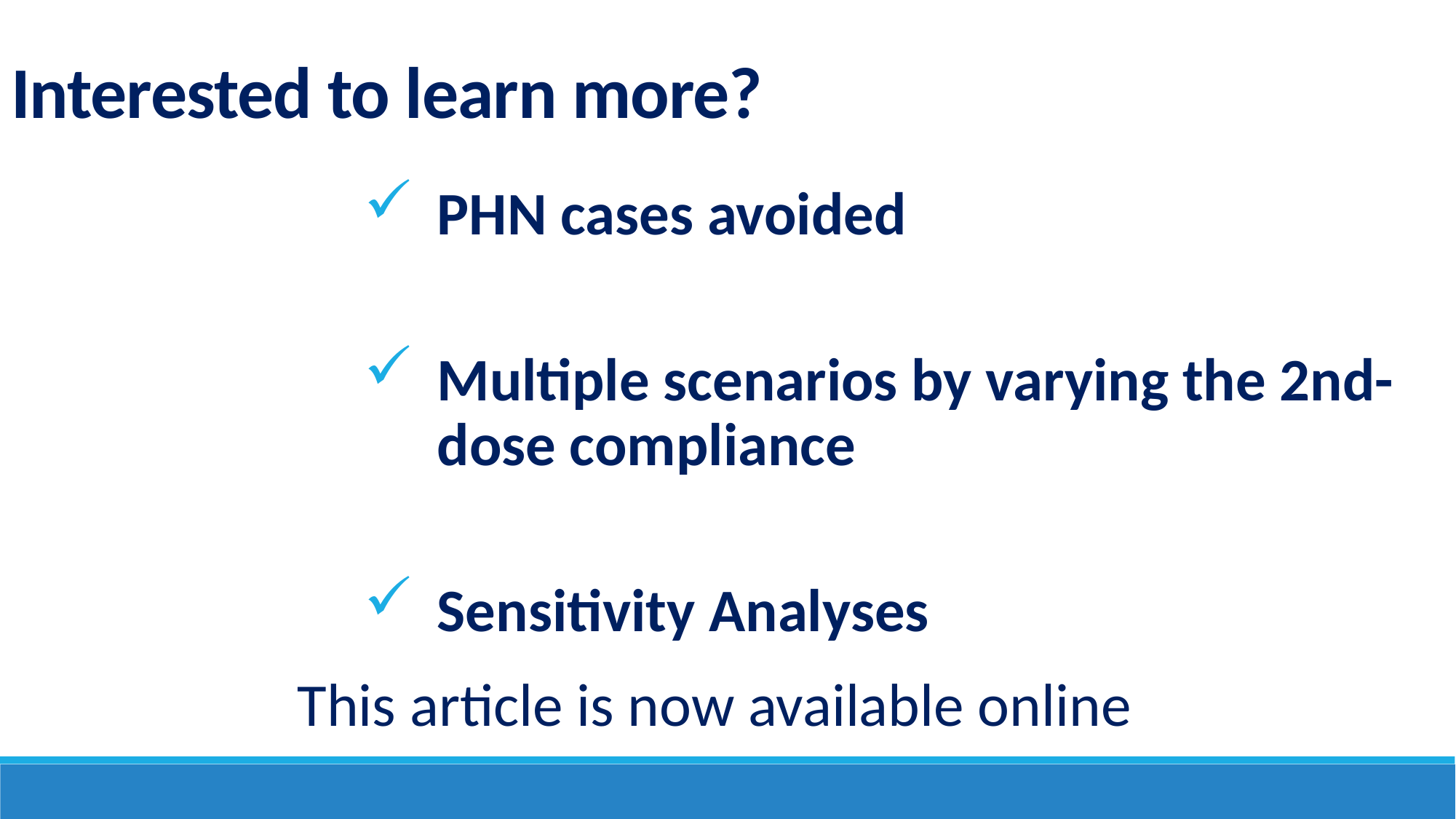

Interested to learn more?
PHN cases avoided
Multiple scenarios by varying the 2nd-dose compliance
Sensitivity Analyses
This article is now available online

## Slide 18
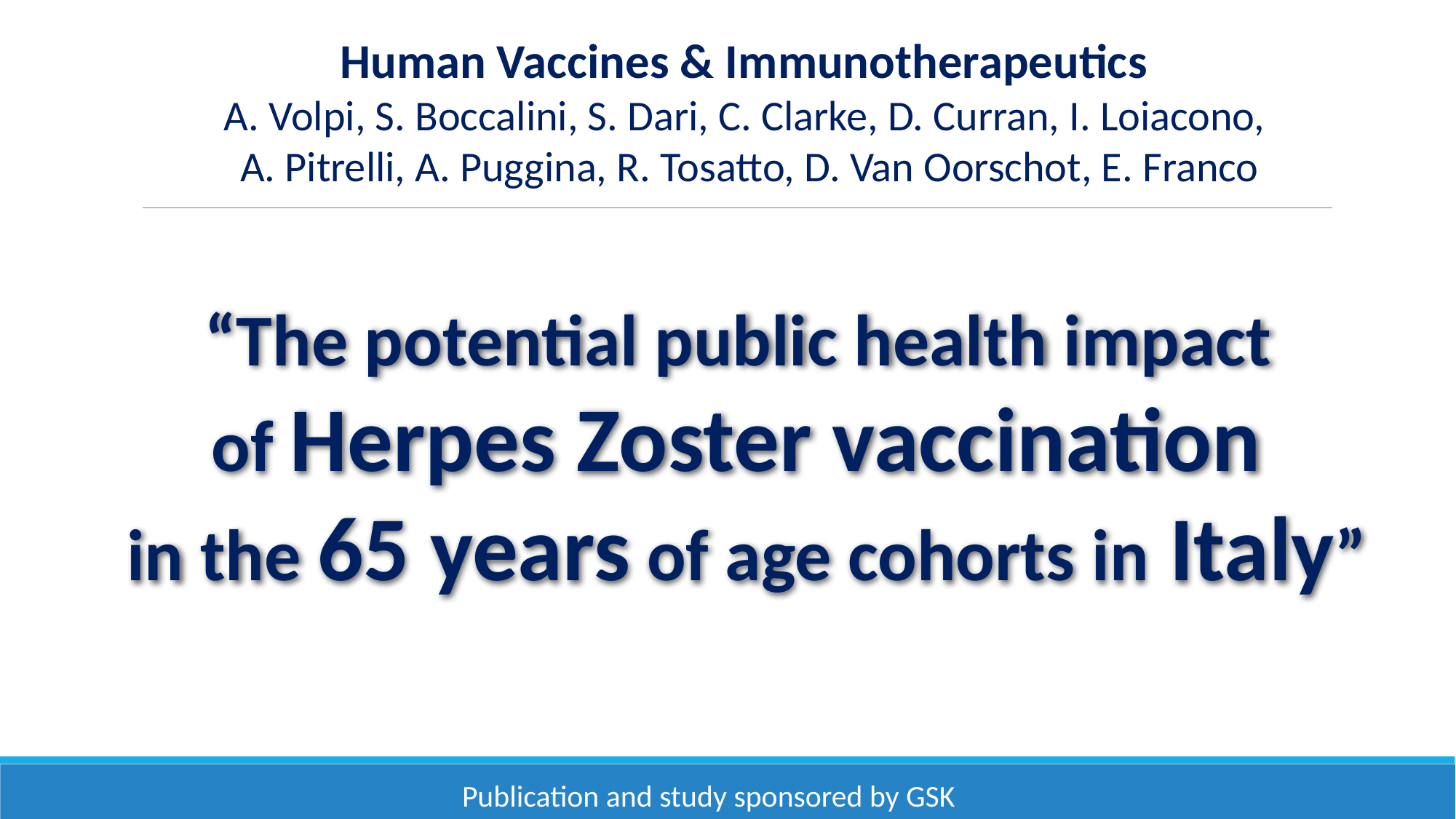

Human Vaccines & Immunotherapeutics
A. Volpi, S. Boccalini, S. Dari, C. Clarke, D. Curran, I. Loiacono,
A. Pitrelli, A. Puggina, R. Tosatto, D. Van Oorschot, E. Franco
“The potential public health impact
of Herpes Zoster vaccination
in the 65 years of age cohorts in Italy”
Publication and study sponsored by GSK
