# Supplementary material for: The potential public health impact of Herpes Zoster vaccination in the 65 years of age cohort in Italy
Source: Hum Vaccin Immunother. 2019 Sep 24;16(2):327–34. doi: 10.1080/21645515.2019.1657753 (PMC7062451; doi:10.1080/21645515.2019.1657753)
Supplement: Supplemental Material [file khvi-16-02-1657753-s001.zip › Supplementary Material - cleaned.docx]

**The potential public health impact of Herpes Zoster vaccination in the 65 years of age cohort in Italy**

**Supplementary material**

**Table S1.** Vaccine efficacy against HZ and PHN.

| **Variable** | **Base-case scenario** | **Source** |
| --- | --- | --- |
| **Vaccine Efficacy Data*** | | |
| ***RZV*** | | |
| Vaccine efficacy (2 doses, 65 YOA) | 98.4% | Curran et al.^1^ |
| Waning (2 doses)** |  | Curran et al.^1^ |
| 65-69 YOA (1-4 years after vaccination) | 1.0% |  |
| 65-69 YOA (≥4 years after vaccination) | 2.3% |  |
| ≥70 YOA | 3.6% |  |
| Vaccine efficacy (1 dose, 65 YOA) | 90.0% | Curran et al.^1^ |
| Waning (1 dose) ** |  | Le et al.^2^ |
| 1-4 years after vaccination | 10.9% |  |
| ≥4 years after vaccination | 10.9% |  |
| ***ZVL*** | | |
| Vaccine efficacy against HZ (65YOA) | 63.9% | Oxman et al.^3^ |
| Vaccine efficacy against PHN (65YOA) | 65.7% | Oxman et al.^3^ |
| Waning** |  | Oxman et al.,^3^ Schmader et al.,^4^ Morrison et al.^5^ |
| 1-4 years after vaccination | 5.4% |  |
| ≥4 years after vaccination | 5.1% |  |

*Initial vaccine efficacy at time 0;

**Waning (absolute percentage) over time of efficacy in preventing HZ;

YOA: years of age; HZ: Herpes Zoster; LTPS: Long-Term Persistence Study; PHN: postherpetic neuralgia; RZV: Adjuvanted Recombinant Zoster Vaccine; SPS: Shingles Prevention Study; STPS: Short-Term Prevention Study; ZVL: Zoster vaccine Live.

**Table S2.** Input variables for the Deterministic Sensitivity Analysis.

| **Variable** | **Low Estimate** | **High Estimate** |
| --- | --- | --- |
|  | | |
| **HZ Incidence (HZ/1,000 person-years)** | Alicino et al.^6^ | Coretti et al.^7^ +20% |
| 65-69 YOA | 6.07 | 8.52 |
| 70-79 YOA | 7.86 | 10.08 |
| ≥80 YOA | 7.86 | 10.08 |
|  | | |
| **Vaccine Coverage at Year 1** | Assumption | Assumption |
|  | 10% | 40% |
|  | | |
| **RZV Second-Dose Compliance** | Assumption | Assumption |
|  | 50% | 90% |
|  | | |
| ***RZV*** | | |
| Vaccine efficacy (2 doses, 65 YOA) | Curran et al.^1^ | Curran et al.^1^ |
|  | 95% | 100% |
|  | | |
| Waning (2 doses)* | Curran et al.^1^ | Curran et al.^1^ |
| 65-69 YOA (1-4 years after vaccination) | 0% | 2.6% |
| 65-69 YOA (≥4 years after vaccination) | 0.7% | 4.6% |
| ≥70 YOA | 1.4% | 6.6% |
|  | | |
| Vaccine efficacy (1 dose, 65 YOA) | Curran et al.^1^ | Curran et al.^1^ |
|  | 58.9% | 98.9% |
|  | | |
| Waning (1 dose)* | Assumption | Assumption |
| 1-4 years after vaccination | 5.5% | 16.4% |
| ≥4 years after vaccination | 5.5% | 16.4% |
|  | | |
| ***ZVL*** | | |
| Vaccine efficacy (65 YOA) | Prescribing Information^@,8^ | Prescribing Information^@,8^ |
|  | 56% | 71% |
|  | | |
| Waning* | Curran et al.^1^ | Curran et al.^1^ |
| 1-4 years after vaccination | 4.5% | 6.4% |
| ≥4 years after vaccination | 4.1% | 6.0% |

* Waning over time of efficacy in preventing HZ;

YOA: years of age; HZ: Herpes Zoster; RZV: Adjuvanted Recombinant Zoster Vaccine; ZVL: Zoster Vaccine Live. @: data from the SPS study, reported in the Prescribing Information (PI).^10^

**Table S3.** Public health impact of RZV and ZVL in the 65 years of age Italian cohort at Year 2, assuming a coverage rate of 35% over a lifetime horizon from the date of vaccination and different RZV second-dose compliance rates.

|  | **Cohort 2 - Year 2** | | | | | | | | | | | |
| --- | --- | --- | --- | --- | --- | --- | --- | --- | --- | --- | --- | --- |
| Individuals 65 YOA^9^ | 762,408 | | | | | | | | | | | |
| Vaccine coverage^10^ and corresponding population^#^ | 35% | | 266,843 | | 35% | | 266,843 | | 35% | | 266,843 | |
| RZV second-dose compliance and corresponding population^§^ | 50% | | 133,421 | | 70% | | 186,790 | | 90% | | 240,159 | |
| ***Outcomes*** | **RZV**  **vs**  **no vaccination** | **ZVL**  **vs**  **no vaccination** | | **RZV**  **vs**  **ZVL** | **RZV**  **vs**  **no vaccination** | **ZVL**  **vs**  **no vaccination** | | **RZV**  **Vs**  **ZVL** | **RZV**  **vs**  **no vaccination** | **ZVL**  **vs**  **no vaccination** | | **RZV**  **vs**  **ZVL** |
| **Clinical impact of HZ vaccination** | | | | | | | | | | | | |
| HZ cases avoided | 17,086 | 9,284 | | 7,802 | 21,148 | 9,284 | | 11,864 | 25,210 | 9,284 | | 15,926 |
| PHN cases avoided | 3,874 | 2,151 | | 1,723 | 4,843 | 2,151 | | 2,692 | 5,812 | 2,151 | | 3,662 |
| Complications avoided | 1,435 | 780 | | 655 | 1,776 | 780 | | 997 | 2,118 | 780 | | 1,338 |
| HZ-related deaths avoided | 3 | 1 | | 2 | 4 | 1 | | 3 | 5 | 1 | | 4 |
| **Economic impact* of HZ vaccination** | | | | | | | | | | | | |
| Direct costs avoided (€) | 4,050,387 | 2,392,147 | | 1,658,240 | 4,941,251 | 2,392,147 | | 2,549,105 | 5,832,115 | 2,392,147 | | 3,439,969 |
| Indirect costs avoided (€) | 4,581,361 | 3,392,693 | | 1,188,669 | 4,951,145 | 3,392,693 | | 1,558,453 | 5,320,930 | 3,392,693 | | 1,928,237 |

YOA: years of age; HZ: Herpes Zoster; PHN: postherpetic neuralgia; RZV: Adjuvanted Recombinant Zoster Vaccine; ZVL: Zoster Vaccine Live.

^#^Receiving ZVL or the first dose of RZV. ^§^Receiving the second dose of RZV. *Related to the cost savings derived from the clinical impact.

**Table S4.** Public health impact of RZV and ZVL in the 65 years of age Italian cohort at Year 3, assuming a coverage rate of 50% over a lifetime horizon from the date of vaccination and different RZV second-dose compliance rates.

|  | **Cohort 3 - Year 3** | | | | | | | | | | | |
| --- | --- | --- | --- | --- | --- | --- | --- | --- | --- | --- | --- | --- |
| Individuals 65 YOA^9^ | 778,463 | | | | | | | | | | | |
| Vaccine coverage^10^ and corresponding population^#^ | 50% | | 389,232 | | 50% | | 389,232 | | 50% | | 389,232 | |
| RZV second-dose compliance and corresponding population^§^ | 50% | | 194,616 | | 70% | | 272,462 | | 90% | | 350,308 | |
| ***Outcomes*** | **RZV**  **vs**  **no vaccination** | **ZVL**  **vs**  **no vaccination** | | **RZV**  **vs**  **ZVL** | **RZV**  **vs**  **no vaccination** | **ZVL**  **vs**  **no vaccination** | | **RZV**  **Vs**  **ZVL** | **RZV**  **vs**  **no vaccination** | **ZVL**  **vs**  **no vaccination** | | **RZV**  **vs**  **ZVL** |
| **Clinical impact of HZ vaccination** | | | | | | | | | | | | |
| HZ cases avoided | 24,923 | 13,542 | | 11,381 | 30,848 | 13,542 | | 17,306 | 36,772 | 13,542 | | 23,230 |
| PHN cases avoided | 5,651 | 3,137 | | 2,513 | 7,064 | 3,137 | | 3,927 | 8,478 | 3,137 | | 5,341 |
| Complications avoided | 2,094 | 1,138 | | 956 | 2,591 | 1,138 | | 1,454 | 3,089 | 1,138 | | 1,951 |
| HZ-related deaths avoided | 4 | 1 | | 3 | 6 | 1 | | 5 | 7 | 1 | | 6 |
| **Economic impact* of HZ vaccination** | | | | | | | | | | | | |
| Direct costs avoided (€) | 5,908,116 | 3,489,316 | | 2,418,800 | 7,207,579 | 3,489,316 | | 3,718,263 | 8,507,042 | 3,489,316 | | 5,017,727 |
| Indirect costs avoided (€) | 6,682,624 | 4,948,767 | | 1,733,857 | 7,222,012 | 4,948,767 | | 2,273,245 | 7,761,399 | 4,948,767 | | 2,812,632 |

YOA: years of age; HZ: Herpes Zoster; PHN: postherpetic neuralgia; RZV: Adjuvanted Recombinant Zoster Vaccine; ZVL: Zoster Vaccine Live.

^#^Receiving ZVL or the first dose of RZV. ^§^Receiving the second dose of RZV. *Related to the cost savings derived from the clinical impact.

**References**

1. Curran D, Van Oorschot D, Varghese L, Oostvogels L, Mrkvan T, Colindres R, von Krempelhuber A, Anastassopoulou A. Assessment of the potential public health impact of Herpes Zoster vaccination in Germany. Hum Vaccin Immunother. 2017;13(10):2213-2221. doi:10.1080/21645515.2017.1345399.

2. Le P, Rothberg MB. Cost-effectiveness of the adjuvanted herpes zoster subunit vaccine in older adults. JAMA Intern Med. 2018;178(2):248-258. doi:10.1001/jamainternmed.2017.7431.

3. Oxman MN, Levin MJ, Johnson GR, Schmader KE, Straus SE, Gelb LD, Arbeit RD, Simberkoff MS, Gershon AA, Davis LE, et al. A Vaccine to Prevent Herpes Zoster and Postherpetic Neuralgia in Older Adults. N Engl J Med. 2005;352(22):2271-2284. doi:10.1056/NEJMoa051016.

4. Schmader KE, Oxman MN, Levin MJ, Johnson G, Zhang JH, Betts R, Morrison VA, Gelb L, Guatelli JC, Harbecke R, et al. Persistence of the Efficacy of Zoster Vaccine in the Shingles Prevention Study and the Short-Term Persistence Substudy. Clin Infect Dis. 2012;55(10):1320-1328. doi:10.1093/cid/cis638.

5. Morrison VA, for the Shingles Prevention Study G, Johnson GR, for the Shingles Prevention Study G, Schmader KE, for the Shingles Prevention Study G, Levin MJ, for the Shingles Prevention Study G, Zhang JH, for the Shingles Prevention Study G, et al. Long-term Persistence of Zoster Vaccine Efficacy. Clin Infect Dis. 2015;60(6):900-909. doi:10.1093/cid/ciu918.

6. Alicino C, Trucchi C, Paganino C, Barberis I, Boccalini S, Martinelli D, Pellizzari B, Bechini A, Orsi A, Bonanni P, et al. Incidence of herpes zoster and post-herpetic neuralgia in Italy: Results from a 3-years population-based study. Hum Vaccin Immunother. 2017;13(2):399-404. doi:10.1080/21645515.2017.1264834.

7. Coretti S, Codella P, Romano F, Ruggeri M, Cicchetti A. Cost-Effectiveness Analysis of Herpes Zoster Vaccination in Italian Elderly Persons. Int J Technol Assess Health Care. 2016;32(4):233-240. doi:10.1017/S0266462316000337.

8. Merck & Co. Inc. Zostavax (zoster vaccine live) prescribing information. Available from [www.merck.com/product/usa/pi_circulars/z/zostavax/zostavax_pi2.pdf](file:///C:\Users\ld123361\AppData\Local\Microsoft\Windows\Temporary%20Internet%20Files\Content.Outlook\6SW1ZEWS\www.merck.com\product\usa\pi_circulars\z\zostavax\zostavax_pi2.pdf). [Accessed June 15, 2017].

9. Istat - Istituto Nazionale di Statistica. Statistiche demografiche. Available from <http://demo.istat.it/pop2017/index.html>. [Accessed July 23, 2018].

10. Gazetta Ufficiale della Republica Italiana. Piano Nazionale Prevenzione Vaccinale 2017-2018 (PNPV), 2017. Available from <http://www.gazzettaufficiale.it/eli/id/2017/02/18/17A01195/sg>. [Accessed July 23, 2018].
